# APETALA2 control of barley internode elongation

**DOI:** 10.1242/dev.170373

**Published:** 2019-06-12

**Authors:** Vrushali Patil, Hannah I. McDermott, Trisha McAllister, Michael Cummins, Joana C. Silva, Ewan Mollison, Rowan Meikle, Jenny Morris, Pete E. Hedley, Robbie Waugh, Christoph Dockter, Mats Hansson, Sarah M. McKim

**Affiliations:** 1 Division of Plant Sciences, School of Life Sciences, The University of Dundee at The James Hutton Institute, Invergowrie, Dundee DD2 5DA, Scotland; 2 Cell and Molecular Sciences, The James Hutton Institute, Invergowrie, Dundee DD2 5DA, Scotland; 3 Carlsberg Research Laboratory, J.C. Jacobsens Gade 4, DK-1799 Copenhagen V, Denmark; 4 Department of Biology, Lund University, Sölvegatan 35B, 22362 Lund, Sweden

**Keywords:** APETALA2, Jasmonate, Phase change, Cereal development, Intercalary meristem, Stem elongation

## Abstract

Many plants dramatically elongate their stems during flowering, yet how this response is coordinated with the reproductive phase is unclear. We demonstrate that microRNA (miRNA) control of *APETALA2* (*AP2*) is required for rapid, complete elongation of stem internodes in barley, especially of the final ‘peduncle’ internode directly underneath the inflorescence. Disrupted miR172 targeting of *AP2* in the *Zeo1.b* barley mutant caused lower mitotic activity, delayed growth dynamics and premature lignification in the peduncle leading to fewer and shorter cells. Stage- and tissue-specific comparative transcriptomics between *Zeo1.b* and its parent cultivar showed reduced expression of proliferation-associated genes, ectopic expression of maturation-related genes and persistent, elevated expression of genes associated with jasmonate and stress responses. We further show that applying methyl jasmonate (MeJA) phenocopied the stem elongation of *Zeo1.b*, and that *Zeo1.b* itself was hypersensitive to inhibition by MeJA but less responsive to promotion by gibberellin. Taken together, we propose that miR172-mediated restriction of *AP2* may modulate the jasmonate pathway to facilitate gibberellin-promoted stem growth during flowering.

## Abstract (precis)

**Summary:** Regulation of reproductive stem elongation in barley by APETALA2 suggests a pivotal role for phase change repression of JA-associated responses to promote internode growth.

## INTRODUCTION

Plants undergo profound changes in architecture during post-embryonic growth owing to altered activity within the shoot apical meristem (SAM) at the stem tip. In the vegetative phase, the SAM sequentially adds body parts to the vertical shoot axis, producing a stem of alternating leafy nodes and internodes (Bell and Bryan, 2008; Galinat, 1959; Gray, 1879; Sharman, 1942). Over time and in response to external and internal cues, the SAM stops making leaves and instead makes a flowering inflorescence, marking the start of the reproductive phase. Many plants respond to flowering by rapidly elongating existing vegetative internodes and new reproductive internodes, displacing the flowering tip upwards on a long stem (Bell and Bryan, 2008). Although networks directing the SAM floral transition have received considerable attention (Wils and Kaufmann, 2017), how reproductive stem elongation is coordinated with flowering remains understudied (McKim, 2019).

Regulation of proliferation and expansion in specific cell populations is crucial for stem morphogenesis. Stem growth in most plants, including the model plant *Arabidopsis*, derives from the subapical rib meristem, where oriented cell division leads to basipetal differentiation of stem tissues, activity of which increases during flowering (Bencivenga et al., 2016; Ruonala et al., 2008; Sachs, 1965). However, many grasses, including cereals, show an additional strategy whereby proliferation within intercalary meristems or division zones found at the base of each internode displaces cells acropetally into an overlying expansion zone where they subsequently expand and transit into an apical maturation zone (Bleecker et al., 1986; Cho and Kende, 1997; Evans, 1965; Fisher, 1970; Fisher and French, 1976; Martin et al., 2016; Schmalfuß, 1930). Gibberellin (GA) phytohormones promote both types of vertical stem growth through increasing cell division and cell expansion (Kende et al., 1998; Sachs, 1965). Higher yielding semi-dwarf rice and wheat of the Green Revolution are impaired in GA biosynthesis or perception, respectively (Hedden, 2003; Peng et al., 1999; Webb et al., 1998). Brassinosteroids (BR) are also important positive regulators of stem growth, with biosynthetic and signalling mutants showing semi-dwarfism (Dockter et al., 2014). Phytohormones also interact to control stem growth, as in the modulation of GA signalling and sensitivity by ethylene and BR during flood-induced stem elongation in rice (Loreti et al., 2016; Schmitz et al., 2013). However, how phytohormones or other regulators synchronise rapid stem growth with the reproductive phase is less understood.

Phase transitions in plants are regulated by deeply conserved antagonism between microRNA156 (miR156), associated with juvenility, and miR172, linked to adult or reproductive fates, and their target transcripts encoding the SQUAMOSA PROMOTER BINDING LIKE (SPL) and APETALA2-like (AP2-like) transcription factors, respectively (Huijser and Schmid, 2011; Poethig, 2013). Our previous work showed that the compact inflorescences of the classic *Zeocriton* (*Zeo*) mutants of barley are caused by gain-of-function mutations in *HvAPETALA2* (*HvAP2*) that delay spike differentiation after the reproductive transition. The causal mutations are associated with disrupted miR172 targeting, leading to higher transcript levels (Houston et al., 2013) that may also influence protein levels (Anwar et al., 2018). *Zeo* mutants are also reported to be short (Franckowiak and Lundqvist, 2011), suggesting a possible link between the phase change miRNA network and reproductive stem elongation. Here, we use comparative developmental and transcriptomic studies between the severe *Zeo1.b* near isogenic line (BW938) and its recurrent parent cultivar (*cv.*) Bowman, as well as hormone sensitivity and genetic analyses, to discover how phase change progression influences internode development. We demonstrate that loss of miRNA regulation of *HvAP2* reduces proliferation and expansion and causes precocious maturation in internodes, which correlates with misexpression of specific proliferation and maturation-related genes. We further reveal that miR172-resistant *HvAP2* elevates jasmonate (JA) and stress-responsive gene expression. Applying methyl jasmonate (MeJA) onto Bowman phenocopied *Zeo1.b* stem elongation and *Zeo1.b* itself showed MeJA hypersensitivity and muted GA responses. We propose that miR172-mediated restriction of *HvAP2* may repress JA-associated pathways in the stem to promote fast, GA-mediated extension during flowering.

## RESULTS

### Disrupted *miR172*-regulation of *HvAP2* leads to fewer and shorter internode cells

**Fig. 1.**
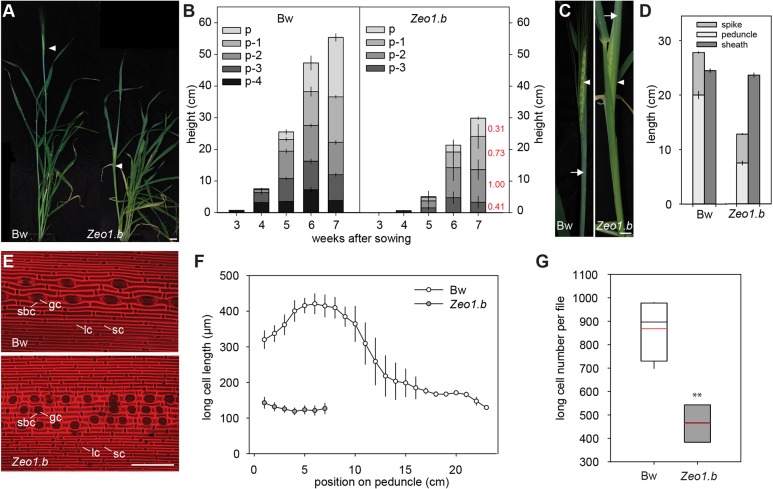
**Internode growth and anatomy.** (A) Glasshouse-grown Bowman (left) and *Zeo1.b* (right) plants at heading. Arrowheads indicate the spike. (B) Internode elongation in Bowman and *Zeo1.b*. Internode labels show position relative to peduncle (p) (*n*=16/genotype). Numbers in red show proportion of each *Zeo1.b* internode's length compared with Bowman at 7 weeks. (C) Emerged Bowman spike (left) and enclosed *Zeo1.b* spike (right). Arrowheads point to spike, arrows to flag leaf sheath. (D) Bowman and *Zeo1.b* peduncle, flag leaf sheath and spike lengths (*n*=17 Bowman; *n*=15 *Zeo1.b*). (E) Propidium iodide-stained epidermis from Bowman (top) and *Zeo1.b* (bottom) peduncles. (F) Average long-cell length (µm) per 1 cm peduncle segment (*n*=3/genotype). (G) Estimated number of long cells per file in Bowman and *Zeo1.b* peduncles. Box plots show 25th to 75th percentiles; whiskers extend down to 10th and up to 90th percentiles; black line shows median; and red line shows mean. Bw, Bowman; gc, guard cell; lc, long cell; sbc, subsidiary cell; sc, silica-cork cell pair. **P=0.004 (Student's *t*-test). Scale bars: 2 cm (A); 1 cm (C); 100 μm (E). Error bars represent s.e.m.

To determine whether *Zeo1.b* semi-dwarfism reflects fewer or shorter internodes or both, we examined each internode of the main stem or ‘culm’, numbered with respect to the peduncle (p) internode directly under the ‘spike’ inflorescence in *Zeo1.b* compared with Bowman (Fig. 1A). Although internodes in both genotypes elongated acropetally, *Zeo1.b* internodes elongated less and more slowly, with the fourth internode below the peduncle (p-4) lacking elongation altogether (Fig. 1B). *Zeo1.b* peduncles were especially stunted reaching only 31% of Bowman length (Fig. 1B), and grew more slowly at 0.31 cm/day compared with 1.5 cm/day in Bowman (Fig. S1). Peduncle elongation pushed the Bowman spike out of the flag leaf sheath (called ‘heading’) whereas short peduncles in *Zeo1.b* left its spike shrouded in the flag leaf sheath (Fig. 1C,D).

Shorter internodes in *Zeo1.b* may derive from fewer and/or shorter cells and/or changes in internode patterning. To examine these possibilities, we analysed peduncle epidermal anatomy. The peduncle epidermis in Bowman comprised uniseriate cell files, of which longitudinally expanded or ‘long’ cells were most abundant and usually alternated in the file with suberized cork cells or silica-cork cell pairs (identified according to Kaufman et al., 1970), and were separated by guard cell and subsidiary cell pairs (stoma) files (Fig. 1E). Measuring long cell lengths along entire peduncles (*n*≥3/genotype) showed that long cells were 5.7-fold longer or ‘hyperelongated’ in the basal segment versus the top in Bowman (Fig. 1F), a gradient absent in other internodes (Fig. S2), and that basal segments lacked the stoma and silica cork cell pairs abundant in the apical region (Fig. S3). *Zeo1.b* had a similar epidermal cell morphology to the Bowman apical peduncle in both cell length and patterning (Fig. 1E,F; Fig. S3). We used the average cell length within each 1 cm segment to estimate cell number per file per 1 cm bin, and then added numbers together to calculate a total number of cells per file along the entire peduncle length. This revealed that *Zeo1.b* had 53% of the total peduncle cells compared with Bowman (*P*=0.004; Fig. 1G), yet because *Zeo1.b* peduncles were 31% of the Bowman length, this additional reduction reflects the loss of the hyperelongated basal cells. Thus, a combination of fewer hyperelongated cells and reduced overall cell number underlies the short peduncles of *Zeo1.b*, indicating that miR172-mediated regulation of *HvAP2* promotes both cell proliferation and basal cell expansion.

### miR172-mediated restriction of *HvAP2* promotes division zone activity and progression of peduncle growth

**Fig. 2.**
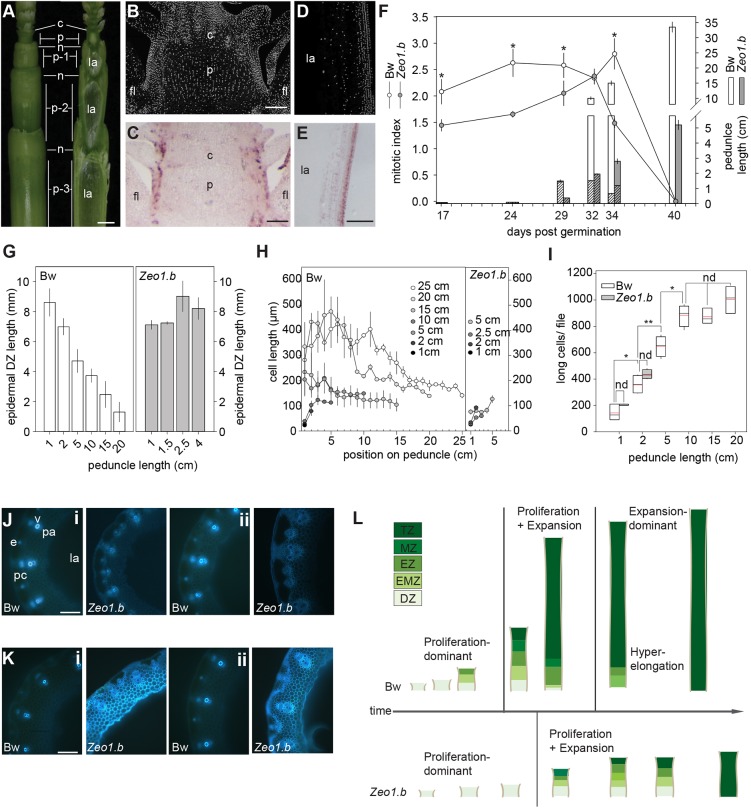
**Developmental origin of *Zeo1.b* semi-dwarfism.** (A) Intact upper barley stem (left) and after sectioning down the middle plane with a razor blade (right). Internodes numbered with respect to the peduncle internode. Leaves removed for clarity. (B-E) DAPI-stained sections (B,D) and *in situ* hybridisation with antisense *HISTONE H4* (*H4*) probe (C,E) through Bowman peduncle initials at 24 days dpg (B,C), and through elongating 1.5 cm Bowman peduncles at 29 dpg (D,E). (F) Lines show average mitotic index of peduncle division zones and bars indicate peduncle length with hatched segments denoting the division zone region (*n*=3/genotype). (G) Proximo-distal length of the epidermal division zones in Bowman and *Zeo1.b* peduncles (*n*=3/genotype). (H) Long cell length (µm) per 1 cm segment of Bowman and *Zeo1.b* growing peduncles at defined lengths (*n*=10 cells/segment/3 biological replicates/genotype). (I) Peduncle cell number per file at defined peduncle lengths during growth (*n*=3/genotype/length). (J,K) Lignin auto-fluorescence in bottom (i) and middle (ii) sections of 2 cm (J) and 5 cm (K) peduncles. (L) Model of peduncle development. Bowman: peduncles entirely proliferative in the first phase, followed by a second phase with distinct division, elongation, maturation and termination zones and a final hyperexpansion phase in the basal peduncle associated with mitotic retreat. *Zeo1.b*: peduncle initiation is delayed, phase progression and growth in the coupled proliferation-expansion phase are slower, and the final hyperexpansion phase is absent. Bw, Bowman; c, collar; DZ, division zone; e, epidermis; EMZ, expansion-maturation transition zone; EZ, expansion zone; fl, flag leaf; la, central lacuna; MZ, maturation zone; n, node; p, peduncle; pa, parenchyma; pc, chlorenchyma; TZ, termination zone; v, vasculature. *P<0.05 (Student's *t*-test). Scale bars: 1 mm (A); 100 µm (B-E,J,K). Error bars represent s.e.m.

In both genotypes, peduncles were initially solid and then formed a central hollow typical of cereal straw as they lengthened (Fig. 2A). Sections stained with 4′,6-diamidino-2-phenylindole (DAPI) and hybridised with antisense *Histone H4* probes showed cell division concentrated in the peripheral cell layers of unexpanded and expanding peduncles (Fig. 2B-E). To determine the origin of the fewer and shorter cells observed in *Zeo1.b*, we examined the activity and size of peduncle division zones. Although Bowman and *Zeo1.b* peduncles at 17 days post-germination (dpg) and 24 dpg were solid, similar lengths (0.50 mm and 0.60 mm long, respectively), and showed cell division throughout, *Zeo1.b* had a lower average mitotic index (MI; percentage of dividing cells/total cell number; *P*<0.05; Fig. 2F). As Bowman peduncles grew to 1.5 cm (29 dpg), proliferation was restricted to the basal 1 cm, marking the division zone, which was maintained as peduncles lengthened to 10 cm (32 dpg). By 15 cm (34 dpg), the division zone retreated to the basal 0.5 cm (*P*<0.05; Fig. 2F). Consistent with slower peduncle elongation (Fig. S1), *Zeo1.b* peduncles at 29 dpg had grown to 2.5 mm long and had a lower mitotic index than Bowman (Fig. 2F). At 32 dpg, the *Zeo1.b* peduncles were 2.5 cm long with a similar 1 cm size and activity of division zone to Bowman (Fig. 2F), but by 34 dpg had a lower mitotic index (*P*<0.05) and lacked any mitotic retreat. The *Zeo1.b* epidermis showed an even more striking loss of mitotic retreat compared with Bowman (Fig. 2G). Thus, miR172-mediated restriction of *HvAP2* appears to be necessary for the rapid generation, proliferative activity and subsequent mitotic retreat of the peduncle division zone.

*Zeo1.b* peduncles also lacked basal elongated cells (Fig. 1F). Examining expansion zone dynamics showed that long cells lengthened up the axis as peduncles grew from 2 cm to 5 cm (Fig. 2H). In Bowman, long cells reached a maximum length mid-way up the 5 cm peduncle (Fig. 2H), and further differentiated acropetally to be highly lignified at the top (Fig. S4), suggesting that cells progressed through expansion to maturation to termination zones. Accordingly, long cell lengths in the top of the Bowman 5 cm peduncle did not elongate further and were as long as cells at the top of fully elongated 25 cm peduncles (*P*=0.43). As Bowman peduncles grew from 10 cm to 15 cm, long cells expanded to a greater extent in the basal regions, suggesting a downward shift of the expansion zone (Fig. 2H). Consistent with division zone regression (Fig. 2F), no further long cells were added to peduncles longer than 15 cm (*P*=0.11; Fig. 2I), indicating that hyperelongation of existing long cells, rather than addition of new cells, drove spike emergence during the last stage of growth (Fig. 2H,I). In contrast, *Zeo1.b* peduncle long cells expanded similarly to apical Bowman peduncles and lacked hyperelongation (Fig. 2H). We also observed that the division and expansion zones of *Zeo1.b* 2 cm peduncles appeared more lignified compared with Bowman 2 cm peduncles harvested 4 days earlier (Fig. 2J), but also compared with 5 cm Bowman peduncles harvested at the same time (Fig. 2K), suggesting that *Zeo1.b* peduncle prematurely lignified. In fact, basal regions of the 5 cm *Zeo1.b* peduncle were entirely lignified (Fig. 2K), potentially explaining the loss of hyperelongation in *Zeo1.b*.

Altogether, our analyses suggest that peduncle development occurs over three phases: an initial proliferation-dominant phase that establishes the division zone; a second, rapid-growth phase that defines a steady-state division zone and overlying expansion, maturation and termination zones; and a final phase of increased longitudinal cell expansion associated with division zone regression (Fig. 2L). Disrupted miR172 targeting of *HvAP2* leads to prolonged, slow growth in the first phase, with lower mitotic activity, followed by a proliferation-expansion phase with a persistent, less active division zone showing maturation features linked to loss of basal cell expansion (Fig. 2L).

### HvAP2 promotes stress signalling in incipient peduncle and spike tissues

**Fig. 3.**
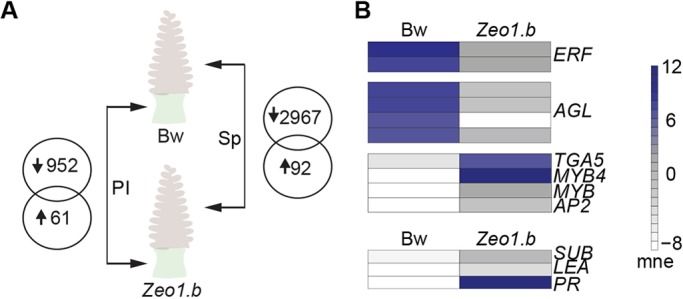
**Comparative transcriptomes of peduncle initials and young spikes.** (A) Venn diagrams show differentially expressed genes in *Zeo1.b* peduncle initials (PI) and spikes (Sp) compared with Bowman (Bw). (B) Heat maps of key genes misregulated in *Zeo1.b* peduncle initials. mne, mean normalised expression; *AGL*, *AGAMOUS-LIKE*; *ERF*, *ETHYLENE RESPONSE FACTOR*; *LEA*, *LATE EMBRYOGENESIS ABUNDANT*; *PR*, *PATHOGENESIS-RELATED*; *SUB*, *SUBTILISIN*.

HvAP2 localises to the nucleus (Fig. S5), where it presumably acts as a transcription factor, suggesting that *Zeo1.b* phenotypes result from HvAP2-mediated changes in gene expression. To test this, we compared gene expression in peduncles and spikes at the start of the first phase of internode elongation (Fig. 3A; Fig. S6A,B). Both these tissues show elevated *HvAP2* expression (Fig. S7; Houston et al., 2013). In *Zeo1.b* versus Bowman, we detected 952 downregulated and 61 upregulated differentially expressed genes (DEGs) in peduncles, and 2967 downregulated and 92 upregulated DEGs in spikes (Table S1A,B). Bowman peduncle initials themselves were enriched for 120 Gene Ontology (GO) terms including cell cycle and DNA replication, whereas DNA biosynthesis and DNA polymerase processes were over-represented in downregulated *Zeo1.b* DEGs (Table S2A-C), consistent with lower mitotic activity in *Zeo1.b*. Bowman spikes were enriched for 133 processes, including shoot system and plant organ development and plastid development, which were also enriched in *Zeo1.b* DEGs (Table S2D,E). Two genes encoding ethylene-responsive factors (ERFs), *ERF1* and *ERF110*, involved in mediating stem elongation in *Arabidopsis* (Zhu et al., 2013), were downregulated in *Zeo1.b* peduncle initials, as were three *AGAMOUS-LIKE* genes (*AGLs*; Fig. 3B; Table S1A), direct targets of AP2 in *Arabidopsis* (Bomblies et al., 1999; Deyholos and Sieburth, 2000; Dinh et al., 2012; Yant et al., 2010), and homologues of *Arabidopsis* genes conferring floral meristem identity (*SEPALLATA3*, *SEP3*; Ditta et al., 2004), flowering and internode growth (*AGL6*; Koo et al., 2010), and phase transitions (*AGL14*; Pérez-Ruiz et al., 2015). Genes encoding a TGA factor normally involved in pathogen defence (Zhang et al., 2003) and a homologue of MYB4, an important mediator of stress and JA responses (Fernández-Calvo et al., 2011; Jin et al., 2000), were upregulated in *Zeo1.b* peduncle initials, as were genes encoding jasmonate-induced proteins (JIPs), thionins and genes associated with stress such as *LATE EMBRYOGENESIS ABUNDANT* (*LEA*), *PATHOGENESIS-RELATED* (*PR*) and *SUBTILISIN* (*SUB*; Fig. 3B; Table S1A). *Zeo1.b* spikes also showed elevated expression of genes encoding a barley orthologue of *Arabidopsis* lipoxygenase 5 (LOX5), chitinase and O-methyltransferase, and those involved in cell wall differentiation (Table S1A). We validated nine DEGs from the peduncle initials and spike microarray by qPCR (Fig. S7). Taken together, *Zeo1.b* showed changes in gene expression consistent with delayed reproductive transition and reduced proliferation, and increased expression of stress-related genes.

### Transcriptomics reveals regulatory modules underlying peduncle developmental zonation

**Fig. 4.**
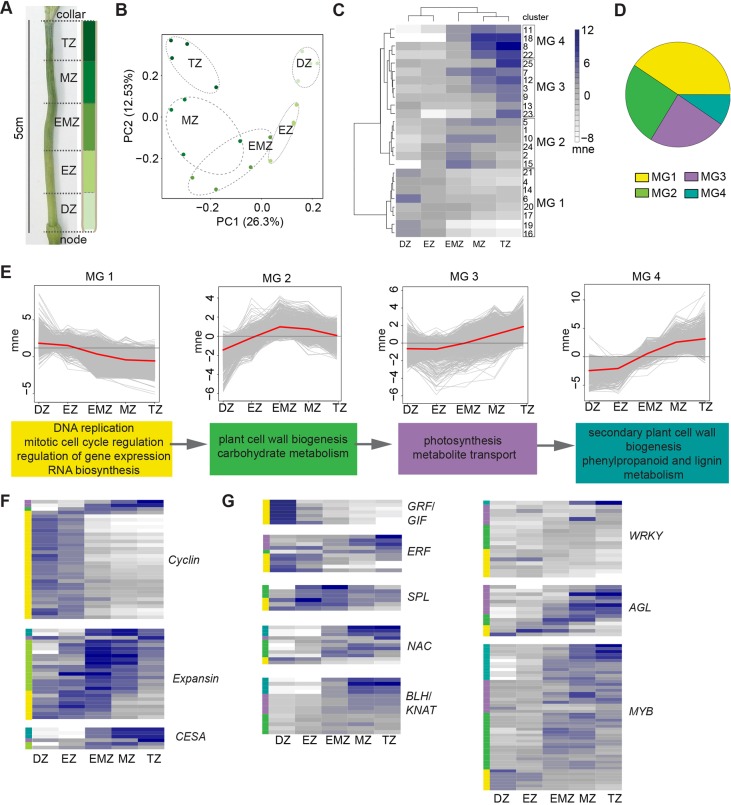
**Elongating peduncle transcriptome.** (A) The 5 cm Bowman peduncle with 1 cm segments classified according to the zonation model. (B) PCA of biological replicates from peduncle segments labelled by zone. (C) Heat map of mean normalised expression of co-expressed clusters showing megaclusters (MGs). (D) Pie chart of differentially expressed genes (DEGs) within each MG. The colour assigned to each MG is consistent throughout the figure. (E) Mean normalised expression profile of each MG in red line. Dark grey line shows mean expression of entire transcriptome and light grey lines show individual DEGs. Colour-coded boxes below each profile show statistically enriched GO terms of the MG. (F,G) Heat maps show mean normalised expression of DEGs encoding cyclins, expansions or cellulose synthases (CESAs) and genes encoding key transcription factor families. Same scale as in C. Coloured bars on the left show the MG assigned to each DEG. Bw, Bowman; DZ, division zone; EZ, expansion zone; EMZ, expansion-maturation transition zone; mne, mean normalised expression; MZ, maturation zone; TZ, termination zone; *ERF*, *ETHYLENE RESPONSE FACTOR*; *GRF*, *GROWTH REGULATING FACTOR*; *GIF*, *GRF INTERACTING FACTOR*; *SPL*, *SQUAMOSA PROMOTER BINDING-LIKE*; *NAC*, *NAM/ATAF/CUC*; *BLH*, *BEL1-LIKE HOMEODOMAIN*; *KNAT*, *KNOTTED-LIKE HOMEODOMAIN*; *AGL*, *AGAMOUS-LIKE*.

To uncover expression differences influenced by elevated *HvAP2* function during the second phase of peduncle elongation, we exploited our internode functional zonation model to examine gene expression in specific internode segments. We first defined the peduncle transcriptome within each 1 cm section along the 5-cm-long elongating Bowman peduncle (Fig. 4A), which corresponded to functional zones as described in Fig. 2L. Adjacent zones were most similar (Fig. 4B), consistent with acropetal transcriptional reprogramming, and 7005 genes were differentially expressed between zones (Table S1C). Hierarchical clustering resolved 25 co-expressed DEG clusters, of which 23 showed statistically significant and often overlapping over-represented GO terms (Fig. 4C; Fig. S8; Table S1C; Table S2F). Using the relationship between the mean expression of each cluster and their GO term overlap, we further hierarchically resolved four ‘megaclusters’ (MGs), with MG 1 having the largest proportion of DEGs (Fig. 4C,D; Table S1C). The MGs represent a collective molecular signature of the developmental gradient with each MG reflecting specific zone activities. Peak expression in MG 1 through to MG 4 shifted from division to termination zones, and GO enrichment correspondingly moved from terms associated with division to expansion and differentiation, and finally to secondary cell wall biosynthesis and lignification (Fig. 4E; Table S2G). DEGs associated with developmental zone functions were represented within the appropriate MG with peak expression in that zone (depicted as bars on the right of heat maps in Fig. 4F; colours match the corresponding MG shown in Fig. 4D; Table S1D). For instance, cyclin-encoding DEGs were preferentially expressed in the division zone and clustered within MG 1, whereas those encoding expansins, cell wall proteins implicated in stem elongation (Cho and Kende, 1997; Marowa et al., 2016), were more highly expressed in the expansion to maturation zone transition and associated with MG 2 (Fig. 4F). Genes encoding cellulose synthases, central to cell wall biosynthesis in *Arabidopsis* (Li et al., 2014) and barley (Burton et al., 2004), were preferentially expressed in expansion to expansion-maturation transition zones, clustering mostly within MG 4 (Fig. 4F), as were phenylpropanoid metabolism genes (Fig. S9). Thus, profiles of key genes and GO enrichment of co-expressed clusters supported that our unbiased clustering resolved biologically relevant gene expression for each functional zone.

To learn more about peduncle regulatory networks, we filtered the peduncle transcriptome for transcription factor (TF)-encoding genes (Fig. 4G). Genes encoding cell proliferation TFs such as GROWTH-REGULATING FACTORS (GRFs) and GRF-INTERACTING FACTORs (GIFs) were highly enriched in the division zone and clustered within MG 1, including homologues encoding GRF5 and GIF1/ANGUSTIFOLIA3 (AN3), direct interaction of which promotes proliferation in leaf primordia (Baute et al., 2015; Horiguchi et al., 2005; Kim and Kende, 2004). Both *GRF5* and *GIF1* are part of MG 1 cluster 6, a cluster almost exclusively expressed in the division zone and uniquely enriched for TF recruitment (Table S2F). In addition, ERFs, such as *ERF1* and *ERF110*, were also highly expressed in the division zone (in MG 1), as were homologues of *AINTEGUMENTA* (*ANT*) and *ANT-like6* (*AIL6*), which redundantly promote cellular proliferation (Krizek, 2009; Mizukami and Fischer, 2000). Genes more highly expressed in expansion and maturation zones (MGs 2-4) included those encoding SPL transcription factors (SPL8/9/13), implicated in stem extension in rice (Wang and Wang, 2015). Similarly, genes encoding homologues of homeodomain proteins, including *BEL1-like1* (*BLH1*), *BLH6*, *BREVIPEDICELLUS* (*BP*) and *KNOTTED-Like7* (*KNAT7*), all of which regulate differentiation kinetics and lignin deposition in *Arabidopsis* and maize (Li et al., 2012, 2014; Liu and Douglas, 2015; Mele et al., 2003; Tsuda et al., 2017), increased in expression towards the maturation and termination zones. A homologue of *BLH4*/*SAW2*, a negative regulator of *BP* in *Arabidopsis* (Kumar et al., 2007), was one of the most highly expressed TFs in the termination zone, which could reflect a transcriptional network to regulate terminal differentiation. Other TF families, such as AGLs, MYBs and WRKYs, were represented by subclusters of DEGs associated with proliferation or expansion/maturation (Fig. 4G). We confirmed the expression pattern of 15 DEGs by qRT-PCR (Fig. S10). Taken together, the elongating peduncle transcriptome identified both mediators and potential upstream regulators of the developmental gradient within the elongating peduncle.

### Disrupted miR172-*HvAP2* interaction causes misexpression of developmental regulators and an elevated expression of JA-associated genes in elongating peduncles

**Fig. 5.**
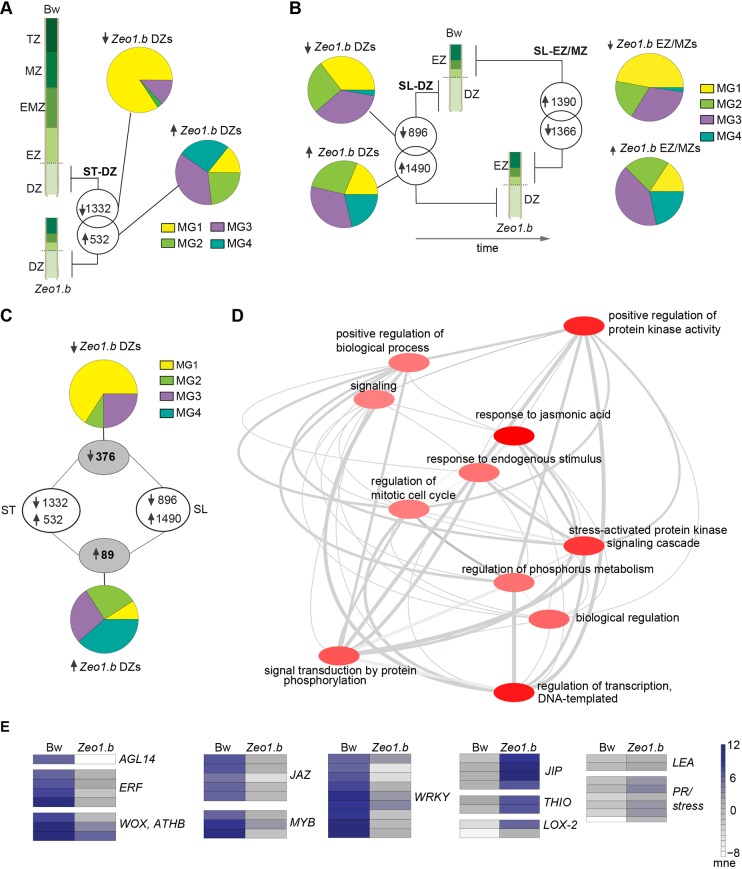
**Comparative transcriptomes of elongating peduncles.** (A) The same time (ST) sampling compared 2 cm *Zeo1.b* and 5 cm Bowman peduncle division zone (DZ) segments harvested at the same time. Venn diagrams show differentially expressed genes (DEGs) in *Zeo1.b* compared with Bowman. Pie charts showing the elongating peduncle transcriptome megacluster (MG) association for down- and upregulated DEGs. (B) The same length (SL) sampling compared 2 cm *Zeo1.b* and 2 cm Bowman peduncle DZ and EZ-MZ segments. Venn diagrams show *Zeo1.b* DEGs and pie charts showing their MG associations. (C) Numbers in grey circles show shared DEGs between DZs of the ST and SL samplings and their MG association. (D) Interaction network of over-represented GO processes in the shared DZ DEGs. The darker the bubble colour, the lower the *P*-value. (E) Heat maps of transcription factors and stress-responsive DEGs. Bw, Bowman; DZ, division zone; EZ, expansion zone; EMZ, expansion-maturation transition zone; mne, mean normalised expression; MZ, maturation zone; TZ, termination zone; *AGL*, *AGAMOUS-LIKE*; *ATHB*, *ARABIDOPSIS THALIANA HOMEOBOX PROTEIN*; *ERF*, *ETHYLENE RESPONSE FACTOR*; *WOX*, *WUSCHEL-like homeobox*; *JAZ*, *JASMONATE INTERACTING FACTORS*; *JIP*, *JAMONATE INDUCED PROTEIN*; *THIO*, *THIONIN*; *LOX2*, *LIPOXYGENASE2*; *LEA*, *LATE EMBRYO ABUNDANT*; *PR*, *PATHOGENESIS-RELATED*.

To determine how HvAP2 may influence the elongating peduncle transcriptome, we first compared gene expression in the lower 1 cm from both 5 cm Bowman and 2 cm *Zeo1.b* peduncles harvested at the same time (ST), which our analyses showed is dividing tissue in both genotypes (Fig. 2F,G). We detected 1332 downregulated and 532 upregulated DEGs in *Zeo1.b* versus Bowman division zones (Fig. 5A; Table S1E). To learn how these DEGs relate to internode zonation, we examined the *Zeo1.b* versus Bowman DEGs for overlap with the MG genes resolved in Fig. 4 (Fig. 5A; Table S1E). The highest proportion of downregulated DEGs with MG association were assigned to MG 1, whereas the upregulated DEGs were more commonly associated with MG 3 and MG 4 (Fig. 5A), consistent with reduced proliferation and precocious differentiation in *Zeo1.b*. Although the small number of shared upregulated DEGs in *Zeo1.b* division zones precluded GO enrichment analysis, downregulated DEGs in *Zeo1.b* division zones were enriched for mitotic cell cycle and daughter terms, including regulation of mitotic cell cycle, regulation of transcription and gene expression and RNA biosynthetic process (Table S2H). Intriguingly, ‘response to jasmonic acid’, a GO term not highlighted in the elongating peduncle transcriptome, was over-represented in the downregulated DEGs, potentially suggesting a stress response in *Zeo1.b* division zones. However, given the differences in peduncle lengths, we reasoned that some DEGs would emerge during a comparison between 5 cm and 2 cm peduncles irrespective of genotype. To address this, we compared 2 cm Bowman and *Zeo1.b* peduncles (same length, SL; Fig. 5B); *Zeo1.b* tissues were older owing to their slower development. Each 2 cm peduncle was sliced into equivalent 1 cm sections, the lower segment comprising proliferating division zone tissue, and the upper 1 cm with cells expanding and maturing, labelled as the EZ-MZ. The division zone and EZ-MZ DEGs in this comparison also showed a higher proportion of downregulated DEGs assigned to MG1 compared with upregulated DEGs, which showed more association with MG3 and MG4 (Fig. 5B; Table S1F,H). GO enrichment of the SL sampling was similar to that of ST (Table S2I,J). The SL comparisons will likely filter both differences due to time and differences due to genotype, so we determined DEGs common to both ST and SL samplings of the division zone, and detected 376 downregulated and 89 upregulated robust DEGs between *Zeo1.b* and Bowman division zones, which showed similar MG associations as before (Fig. 5C; Table S1H). Downregulated DEGs in *Zeo1.b* division zones were enriched for regulation of mitotic cell cycle and regulation of transcription (Fig. 5D; Table S2K), and included multiple genes encoding proliferation-related TF homologues such as WUSCHEL-HOMEOBOX2 (WOX2), GRF5 and GIF3 (Table S1H,I; Fig. 5E). Also downregulated were *ERF1*, *ERF110* and *AGL14* (Table S1H,I). Comparing the EZ-MZs also revealed that *Zeo1.b* was deficient in expression of multiple MG1 genes, such as *GRF5* and *AGL14*, and had regulation of transcription and RNA biosynthetic processes over-represented whereas genes associated with growth repression, such as *LAX.A* (*HvBOP2*), which represses rachis internode elongation in the barley spike (Jost et al., 2016) were upregulated (Tables S1G, S2J). The small number of shared upregulated DEGs in *Zeo1.b* division zones precluded GO enrichment tests, but upregulated DEGs in the same length microarray were enriched for cell wall terms, and included homologues of *Arabidopsis BLH1* and *BLH6* (Tables S1G, S2J).

‘Response to jasmonic acid’ was the most statistically enriched category for shared downregulated DEGs in the *Zeo1.b* division zone (Fig. 5D), mainly reflecting severe reduction in genes encoding JASMONATE-ZIM DOMAIN (JAZ) repressors (Fig. 5E; Table S1E,F,H,I), which are negative regulators of JA signalling (Pauwels and Goossens, 2011; Thines et al., 2007). In contrast, multiple jasmonate-, disease- and stress-related genes were upregulated within *Zeo1.b* division zones (Fig. 5E), where the most highly expressed DEGs encoded phytases, JIPs, thionins and LOX2, a JA biosynthetc enzyme (Bell et al., 1995) (Table 1). Furthermore, many misregulated TFs in *Zeo1.b* division zones have JA signalling and/or defence roles (Table S1J). For instance, two downregulated DEGs showing homology to *Arabidopsis WRKY41*, which suppresses JA-mediated increases in defence gene expression (Higashi et al., 2008), and one of the most severely downregulated DEGs encoding a homologue of *Arabidopsis WRKY70* (Table S1H), which is repressed by JA signalling and itself suppresses JA-responsive genes (Li et al., 2004). Lastly, DEGs upregulated in the *Zeo1.b* expansion zones showed elevated oxylipin metabolism terms (Table S2J) and elevated expression of *LOX2* (Table S1G) may also indicate higher levels of JA (Dave and Graham, 2012). We reconstructed metabolic pathways for JA biosynthesis in the peduncle (Table S1J; Fig. S11) using BarleyCyc from the Plant Metabolic Network (Schläpfer et al., 2017) based on assigning array probes to HORVU gene models (Mascher et al., 2017; Table S3A), and detected ten JA pathway DEGs from the ST and SL microarray, of which only one, *LOX2*, was common and upregulated across microarrays (Table S1J). Seven DZ DEGs were validated by qPCR (Fig. S7). Taken together, comparative peduncle transcriptomics suggested that JA-related responses and/or metabolism are enhanced in *Zeo1.b*, and misregulation of specific proliferation and differentiation-associated DEGs in *Zeo1.b* support their role in the developmental gradient, all of which may be key targets of phase change pathway to control stem growth.

**Table 1.**
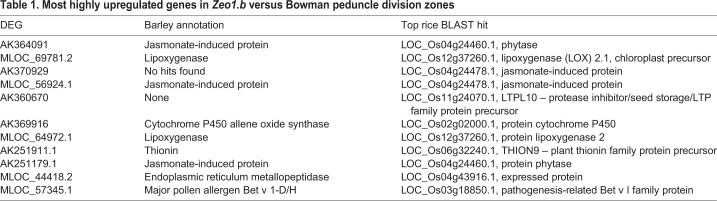
**Most highly upregulated genes in *Zeo1.b* versus Bowman peduncle division zones**

As *HvAP2* was more highly expressed in *Zeo1.b* (Table S1A,B,E,F; Fig. S7), DEGs likely represent a combination of direct and indirect effects of elevated HvAP2. Examining DEG promoters revealed that 172 peduncle initials and 136 shared division zone DEGs contained AP2-binding motif(s) including *WRKY41*, *JAZ*, *ERF7*, *AGL14* and *LOX2* (Table S3B-E). Many of these promoter motifs contained the ‘AAACAA’ consensus AP2-binding motif identified by Dinh et al. (2012) and *LOX2* has been shown to be a direct *Arabidopsis* AP2 target by ChIP-seq (Yant et al., 2010).

### MeJA treatment phenocopies *Zeo1.b* stem elongation

**Fig. 6.**
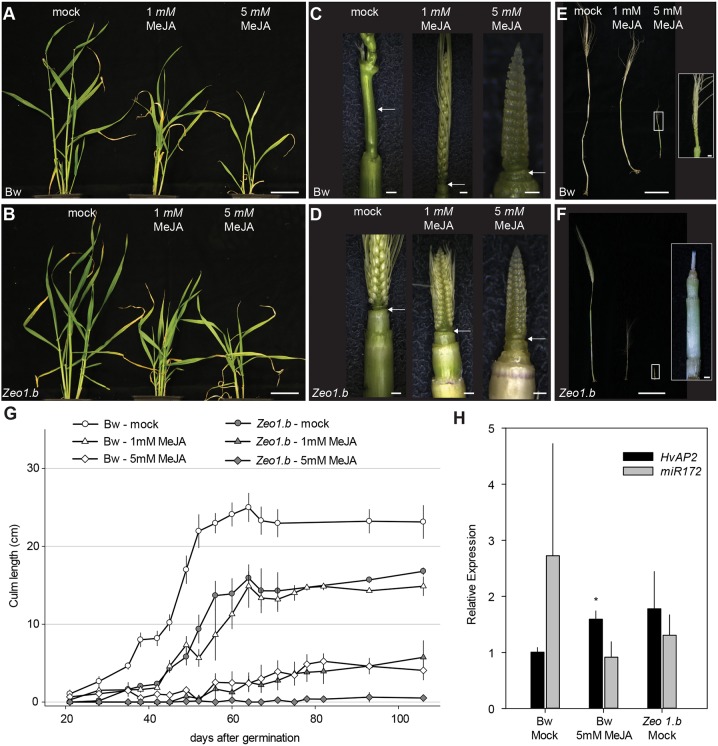
**Methyl jasmonate responses.** (A-F) Plants mock-treated or treated with 1 mM or 5 mM methyl jasmonate (MeJA). (A,B) Bowman (A) and *Zeo1.b* (B) plants at 42 dpg. (C,D) Bowman (C) and *Zeo1.b* (D) spikes and upper culms at 35 dpg. Arrows indicate peduncles. (E,F) Bowman (E) and *Zeo1.b* (F) spikes and culms at 106 dpg; insets show magnification of spikes treated with 5 mM MeJA. (G) Culm elongation in Bowman and *Zeo1.b* treated with mock, 1 mM or 5 mM MeJA (*n*=8/Bowman; *n*=9/*Zeo1.b*). (H) Relative *HvAP2* or miR172 expression in mock or 5 mM MeJA-treated spikes and stem at 35 dpg (*n*=3/genotype). *P=0.02 (Student's *t*-test) between mock- and 5 mM MeJA-treated Bowman. Scale bars: 5 cm (A,B,E,F); 1 mm (C,D, except mock 1 cm); 1 cm (E,F insets). Error bars represent s.e.m.

Transcriptomics pointed to an interaction between elevated HvAP2 function and the JA pathway. To explore this further, we examined the effects of MeJA application. Following a dose-response experiment (Fig. S12), we treated Bowman and *Zeo1.b* plants grown in growth cabinets, with either mock, 1 mM or 5 mM MeJA every 2 days, starting at 14 days after germination after both genotypes had transitioned to spike development (Fig. S13). In Bowman, MeJA treatment caused a dose-responsive delay in stem elongation and spike differentiation with the 5 mM treatment severely dwarfing plants and arresting spike development (Fig. 6A,C,E,G; Fig. S13). A similar but more extreme trend was observed in *Zeo1.b*, with the 5 mM treatment arresting spikes at late awn primordium stage and blocking elongation of almost all internodes (Fig. 6B,D,F; Fig. S13), suggesting that *Zeo1.b* is hypersensitive to MeJA treatment. In both genotypes, MeJA treatment led to decreased long cell number and length (Fig. S13). Glasshouse-grown plants also showed a similar response (Fig. S14). Mock-treated *Zeo1.b* showed slower stem elongation and delayed spike differentiation compared with Bowman, although spikes always fully matured (Fig. 6B,D,F,G). Strikingly, application of 1 mM MeJA to Bowman phenocopied both the rate and extent of stem elongation in mock-treated *Zeo1.b*, and 5 mM MeJA-treated Bowman closely resembled 1 mM MeJA-treated *Zeo1.b* (Fig. 6G). Although variable, we also detected elevated average expression of *HvAP2* (*P*=0.02) and reduction in average expression of miR172 (although not statistically significant; *P*>0.05) in 5 mM MeJA-treated Bowman apices compared with mock tissues, similar to the *Zeo1.b* mock-treated group, suggesting that JA treatment may induce *HvAP2* expression (Fig. 6H). Thus, MeJA-elicited phenotypes support the suggestion that an overactive JA pathway could be a potential mechanism for the inhibition of stem elongation and reproductive phase progression observed in *Zeo1.b*, consistent with its elevated JA-associated gene expression.

### Elevated HvAP2 in *Zeo1.b* leads to reduced sensitivity to gibberellin

**Fig. 7.**
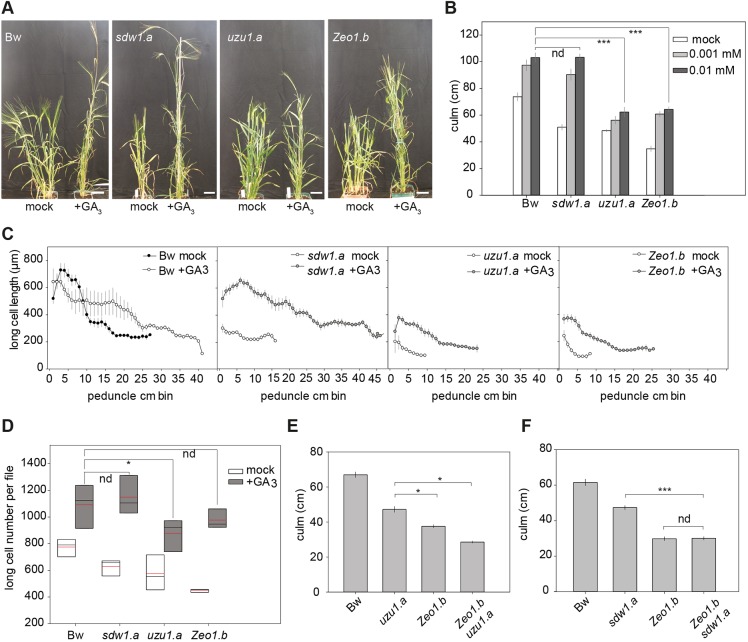
**Gibberellin responses.** (A) Bowman and *Zeo1.b*, *uzu1.a* and *sdw1.a* plants treated with mock or 0.01 mM gibberellin (GA_3_). (B) Culm lengths of Bowman, *sdw1.a*, *uzu1.a* and *Zeo1.b* plants following treatment with mock, 0.001 mM and 0.01 mM GA_3_ (*n*=7-10/genotype/treatment). (C) Long-cell length (µm) per 1 cm segment up the peduncle following either mock or 0.01 mM GA_3_ (*n*=3/genotype/treatment). (D) Long cell number per file in peduncles treated with mock or 0.01 mM GA_3_ (*n*=3 peduncles with 10 cells measured per cm/genotype). Box plots show 25th to 75th percentiles; whiskers extend down to 10th and up to 90th percentiles; black line shows median; and red line shows mean. (E) Culm lengths of Bowman, *uzu1.a*, *Zeo1.b* and *uzu1.a Zeo1.b* plants (*n*=7-19/genotype). (F) Culm lengths of Bowman, *sdw1.a*, *Zeo1.b* and *sdw1.a Zeo1.b* plants (*n*=10/genotype). Bw, Bowman. *P<0.05; ***P<0.001 (Student's *t*-test). nd, not statistically different. Scale bars: 5 cm. Error bars represent s.e.m.

Because both GA and BR pathways promote stem elongation in barley (Dockter and Hansson, 2015) and are themselves targets of JA-mediated growth inhibition (Heinrich et al., 2013; Huang et al., 2017), we explored whether either hormone pathway was compromised in *Zeo1.b*. To examine GA perception and/or signalling, we applied exogenous GA_3_ onto shoots of Bowman and *Zeo1.b* as well as the *sdw1.a* (BW827) mutant, in which the GA biosynthetic *HvGA20ox2* gene is deleted (Xu et al., 2017), and *uzu1.a* (BW885), in which the BR receptor HvBRI1 receptor is impaired (Chono et al., 2003). Treatment concentrations were determined following a dose-response experiment (Fig. S15). Compared with mock treatment, 0.01 mM GA_3_ application increased Bowman culm and peduncle lengths, and rescued *sdw1.a* to the GA_3_-treated Bowman phenotype, and increased the number of long cells per file in proportion to the increase in peduncle length (Fig. 7A-D), showing a primary effect of GA_3_ on proliferation; however, GA_3_ did not rescue either *uzu1.a* or *Zeo1.b* to Bowman-treated lengths (*P*<0.0001, *P*=0.011, respectively; Fig. 7A,B; Fig. S16). Cell number in GA_3_-treated *Zeo1.b* peduncles was equivalent to that of Bowman-treated peduncles (*P*=0.3), suggesting that sufficient GA_3_ restores mitotic activity (Fig. 7D). GA_3_ treatment did not restore the cell-length gradients in either *uzu1.a* or *Zeo1.b* (Fig. 7C), suggesting that *uzu1.a* and *Zeo1.b* may prevent GA-driven cell expansion independently of GA availability, explaining the shorter GA_3_-treated internodes. In *Arabidopsis*, GA promotes cell expansion in part via post-translational control of BR signalling (Bai et al., 2012; Gallego-Bartolome et al., 2012; Li et al., 2012), which, if conserved in barley, may explain the reduced cell expansion in *uzu1.a.* However, compromised BR perception is unlikely to contribute to *Zeo1.b* because BR-induced leaf-segment unrolling (Chono et al., 2003) of *Zeo1.b* and Bowman were equivalent (Fig. S17) and double *Zeo1.b uzu1.a* mutants had shorter culms and peduncles than either parent (*P*=0.05; Fig. 7E; Fig. S18), suggesting both alleles contribute independent effects. In contrast, we detected no difference in length between *Zeo1.b* and *Zeo1.b sdw1.a* culms (*P*=0.8; Fig. 7F) or peduncles (*P*=0.69; Fig. S18), suggesting that *Zeo1.b* is epistatic to *sdw1.a*. Interestingly, the GA biosynthetic gene *HvGA20ox1* was highly upregulated in *Zeo1.b* elongating peduncle division and expansion zones (Table S1E-H; Boden et al., 2014), consistent with feedback sensitivity to reduced GA signalling (Sun, 2008). Taken together, *Zeo1.b* semi-dwarfism is not fully explained by GA deficiency or compromised BR perception, and overexpression of *HvAP2* may additionally inhibit GA-mediated growth responses.

## DISCUSSION

### miR172 targeting of *HvAP2* promotes stem growth after flowering

Internodes and leaves originate from the same group of founder cells off the SAM (Jegla and Sussex, 1989; Johri and Coe, 1983; McDaniel and Poethig, 1988; Poethig et al., 1986; Scanlon et al., 1996). Although both leaves and internodes also undergo a similar developmental programme of proliferation, expansion and maturation (this paper; Nelissen et al., 2016), leaf outgrowth occurs throughout vegetative development, whereas extensive internode growth only occurs after the reproductive transition. This timing may be crucial to ensure that the inflorescence emerges at only reproductive maturity and to establish leaf sheath support for soft internodes developing under an increasingly heavy inflorescence (Haberlandt, 1914). Here, we show that loss of miR172-mediated restriction of *HvAP2* delayed the onset of internode growth throughout the stem, despite the reproductive transition at the apex, and delayed the progression of internode growth phases.

Sequential action of miR156 and miR172 regulates developmental timing across land plants (Poethig, 2009), in which miR172-mediated restriction of *AP2-like* gene function drives differentiation of adult features in vegetative organs, accelerates the reproductive transition and influences floral architecture (Chuck et al., 2008, 2007, 1998; Lee and An, 2012; Mathieu et al., 2009; Moose and Sisco, 1996; Yant et al., 2010; Zhu and Helliwell, 2011). We show that miR172 targeting of *HvAP2* promotes reproductive stem elongation in part through fast generation and subsequent maintenance of a highly active division zone. As the proximo-distal division zone dimensions of *Zeo1.b* were similar to those of Bowman, *HvAP2* effects on the rate and mitotic activity of the division zone is unlikely to influence division zone size. However, comparative lignification assays and transcriptomics imply that the transition between the division, expansion and maturation is less distinct in *Zeo1.b* (Fig. 2; Fig. 5), and that miR172-mediated regulation of *HvAP2* may exclude maturation-related gene expression within proliferating regions. The importance of suppressing maturation programmes was recently demonstrated in maize, in which loss of two homeobox transcription factors caused premature differentiation and shorter internodes (Tsuda et al., 2017).

Although stems grow differently in monocots and dicots, the role of AP2-like transcription factors in suppressing reproductive stem elongation may be conserved as loss of miR172 targeting of the *Arabidopsis* AP2-like *SMZ* or *TOE* also leads to dwarfism (Mathieu et al., 2009) and AP2 proteins directly suppress *AGL* expression to control floral meristem identity and the flowering phase transition (Dinh et al., 2012; Drews et al., 1991; Krogan et al., 2012; Mathieu et al., 2009; Yant et al., 2010). *HvAGLs* downregulated in *Zeo1.b* are potential HvAP2 targets: in particular, the most severely downregulated TF gene encodes a homologue of *Arabidopsis AGL14*, which regulates key transitions, including reproductive stem elongation (Pérez-Ruiz et al., 2015).

### Regulation of JA pathways may control stem elongation

To date, studies on cereal internode growth have either compared different stem internodes (Bosch et al., 2011; Cui et al., 2012; Hu et al., 2017; Kebrom et al., 2017) or focused on specific biological processes within single internodes, such as cell wall or carbohydrate metabolism (Fisher and French, 1976; Lin et al., 2009; Martin et al., 2016; Zhang et al., 2014). Here, we comprehensively characterised the precise spatiotemporal development and transcriptome of initial and elongating peduncles and validated key regulators through comparative study with *Zeo1.b*. This approach identified key developmental regulators misregulated in *Zeo1.b*, such as AGLs and ERFs, but also revealed elevated expression of stress- and JA-related genes from the onset of reproductive internode growth, suggesting that HvAP2 may inhibit stem elongation through pathways typically associated with plant defence. Traditionally interpreted as a direct metabolic trade-off to conserve resources when under threat (Huot et al., 2014), defence-related growth repression may instead activate a JA molecular circuitry that normally controls development independently of stress (Campos et al., 2016). For instance, enhanced JA deactivation or defective JA receptors in *Arabidopsis* and rice are associated with increased overall plant height and longer internodes owing to increased cell number or expansion, respectively (Kurotani et al., 2015; Yang et al., 2012). Elevated JA promotes lignification (Sehr et al., 2010) and increased flux towards JA biosynthesis causes extreme dwarfism and ectopic stem lignification in *Arabidopsis* (Lin et al., 2016). In *Arabidopsis*, overexpression of *BLADE-ON-PETIOLE* (*BOP*) genes led to elevated JA and defence signalling, ectopic lignification, and dwarfism but also misregulation of the miR172-*AP2* network and delayed phase transitions (Khan et al., 2015, 2012). These phenotypes are similar to those of *Zeo1.b* and are consistent with higher levels of *HvLAX.A* (*HvBOP2*) in *Zeo1.b*, which altogether suggest interplay between growth, JA and developmental transitions.

**Fig. 8.**
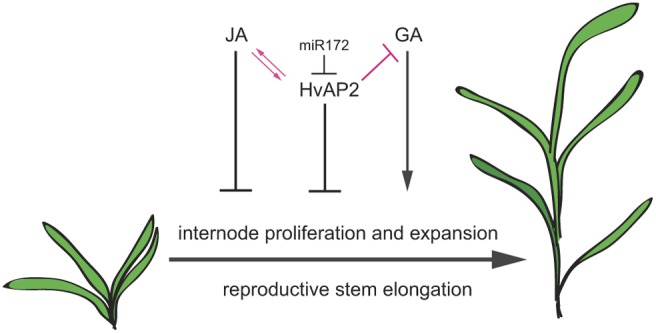
**Hypothetical model of HvAP2 control of stem elongation.** Both HvAP2 and JA inhibit internode cell proliferation and expansion, whereas GA promotes these processes following the reproductive transition. Loss of miR172 targeting of *HvAP2* is associated with JA-associated gene expression, and JA induces *HvAP2* expression, suggesting that HvAP2 and JA may interact to regulate reproductive maturation and stem elongation. Loss of miR172 targeting of *HvAP2* also leads to lower responsiveness to GA-mediated promotion of stem growth. We propose that limiting HvAP2 function via miR172 targeting is important for repression of JA signalling and promotion of the GA-mediated internode elongation in the reproductive phase.

JA promotes juvenility and inhibits the flowering transition in *Arabidopsis* (Song et al., 2013; Yang et al., 2012), a role likely conserved in cereals (this study; Beydler et al., 2016; Hibara et al., 2016), adding to a promising hypothesis that JA levels and/or signalling control multiple morphological and phenological events associated with plant age. For instance, in *Arabidopsis* JA stimulates the release of JAZ repressors bound to AP2-like proteins, leading to delayed flowering time (Zhai et al., 2015) whereas SPL9, a target of miR156, stabilises JAZ3 in older plants to promote phase progression (Mao et al., 2017). Strikingly, we show that MeJA inhibits both developmental transitions and stem elongation, and increases *HvAP2* expression, suggesting a broad conservation of JA-mediated delayed developmental progression. We also found that loss of miR172 targeting of HvAP2 leads to highly elevated expression of *LOX2* and reduced expression of JAZ genes (Fig. 5C), potentially contributing to hypersensitivity to MeJA in *Zeo1.b*, which is also characteristic of *Arabidopsis* multiple *jaz* loss-of-function mutants (Major et al., 2017). JA-mediated growth repression may result in part from interference with the GA pathway. DELLA repressors of GA-activated gene expression are themselves repressed by JAZ protein binding; JAZ degradation in response to JA then releases DELLAs to suppress GA-driven growth (Navarro et al., 2008; Wild et al., 2012). The inability of *Zeo1.b* to phenocopy *sdw1.a* in response to GA application is consistent with elevated DELLA function, which may reflect lower *JAZ* levels in *Zeo1.b*. Here, we show that uncoupling HvAP2 from miR172 represses internode proliferation and expansion, and dampens the internode responsiveness to GA. Our data indicate that both elevated HvAP2 function and exogenous MeJA reduces stem growth by limiting the extent of cell proliferation and expansion within the internode whereas GA promotes both of these processes (Fig. 8, black lines). Our work also shows that loss of miR172 targeting of HvAP2 leads to promotion of JA signalling and muted responses to GA (Fig. 8, pink lines). We propose that miR172 restriction of *HvAP2* may be important to repress JA responses to facilitate the rapid and extensive stem growth promoted by GA during the reproductive phase progression (Fig. 8).

### Agronomic implications

Internode elongation largely determines plant height, a crucial agronomic trait. Stems must be sufficiently strong to support the grain-bearing tip, long enough to reduce susceptibility to soil-borne infections and aid mechanical harvesting, but not so tall that lodging occurs (Rajkumara, 2008). The relationship between internode elongation and yield was famously demonstrated during the Green Revolution by selection of semi-dwarf rice and wheat, later shown to be defective in GA biosynthesis or perception, respectively (Hedden, 2003; Peng et al., 1999; Webb et al., 1998). In barley, impaired GA biosynthesis and BR perception underlies alleles of the two major semi-dwarfing loci, *Sdw1* and *Uzu1.a*, respectively (Chono et al., 2003; Xu et al., 2017). However, the loss-of-function mutation *sdw1* can lead to shortened peduncles and incomplete spike extrusion from the leaf sheath, increasing the risk of grain pathogen attack and the loss of grain yield and quality, and the temperature-sensitivity of *uzu1.a* can prevent peduncle and second internode elongation altogether, leaving the spike stuck within the leaves (Dockter et al., 2014). Recent work revealed that repression of JA metabolism plays a pivotal role in stem elongation in deepwater rice (Minami et al., 2018), a key agronomic feature in flood-prone regions. Our work showcasing roles for JA in reproductive stem elongation suggest that targeting JA metabolism and/or sensitivity may provide additional routes to control cereal height.

## MATERIALS AND METHODS

### Germplasm, growth conditions and basic phenotyping

The cultivar (*cv*.) Bowman and the Bowman Near Isogenic Lines (BWNILs; Druka et al., 2011) *Zeo1.b* (Franckowiak and Lundqvist, 2011; Houston et al., 2013; BW938), *sdw1.a* (BW827) and *uzu1.a* (Chono et al., 2003; Dockter et al., 2014), were grown individually in 5-inch square pots filled with cereal compost under 16 h light and 8 h dark glasshouse conditions maintained between 16 and 24°C with natural light supplemented with high-pressure sodium lamps. Flag leaf sheath was measured from flag leaf attachment at the node to the base of the flag leaf blade. Plants used for MeJA application experiments were grown in controlled environment cabinets (Snijders, NER) under 16 h light (300 µE) and 8 h dark conditions maintained at 18°C. Phenotype data are presented as the mean±s.e.m. along with the number of individuals per sample. Normal distribution was tested with Shapiro–Wilk test for *n*<50 and Lillifors test for *n*>50. Comparisons between two groups were conducted by Student's *t*-test for normal data and Mann–Whitney for non-normal data. Comparisons between more than two groups were conducted by ANOVA (normal data) and Kruskal–Wallis (non-normal data).

### Epidermal cell length measurements

Peduncle tissue was collected from at least three plants per genotype per sampling point, and sectioned into 1 cm segments. Each segment was treated with 100 μg/ml propidium iodide for 1 min for fresh tissue or overnight for dry tissue. The epidermis was imaged with a Nikon A1R confocal microscope using a 560 nm excitation wavelength with a sapphire laser. The longitudinal length of 10-15 long cells at least four cell files away from stomatal files (when present) were measured with Fiji image analysis software (Schindelin et al., 2012). The number of long cells per file per segment was estimated by dividing each segment length (1 cm) by the average cell length, which were then summed to yield the long cell number per file along the internode. For 2 cm developing internodes, the first cm segment consisted of dividing cells and was excluded from calculations of expanding cell number.

### Mitotic index, *in situ* hybridisation, lignin and nuclear localisation

Bowman and *Zeo1.b* peduncle internodes were harvested at 17, 24, 29, 32, 34 and 40 dpg, fixed in 4% paraformaldehyde, processed in a Leica TP1020 Automatic Tissue Processor and embedded in wax using a Leica EG1160 Tissue Embedder and sectioned into 8 μm slices using a Leica MR2265 Fully Motorised Rotary Microtome. Sections were water-mounted on poly-L-lysine-coated slides and heated at 45°C overnight. Prior to tissue staining or *in situ* hybridisation, slides were dewaxed with Histoclear and rehydrated with an ethanol-water series. For mitotic index measurements, dewaxed sections from the centre of the stem axis were stained with DAPI (1 µg/ml) for 30 min in a dark room. Images were captured on a Zeiss LSM 710 confocal microscope under 408 nm laser with tile-scanning. CellProfiler software (Kamentsky et al., 2011) was programmed to count all nuclei and dividing nuclei were manually counted. The division zone was defined as the interval from peduncle base to the last observed dividing cell. At least three images from each biological replicate (*n*=3) were analysed.

For *in situ* hybridisation, dewaxed sections were treated with single-stranded RNA probes transcribed *in vitro* from the PCR-generated DNA templates (see Table S4 for primers) using T7 RNA polymerase (Promega) and digoxygenin-11-UTP-labelled (DIG) nucleotide mix (Sigma) in separate sense (negative control) and antisense orientations with respect to the *Histone4* (*H4*) coding region. Hybridisation and slide washing were adapted from Hooker et al. (2002) and used anti-DIG-AP antibody (11093274910, Roche). Sections were photographed using brightfield optics under Histomount coverslips. Internode tissue was hand-sectioned, mounted in distilled water and visualised under UV light using a 510-560 µm filter on a Nikon compound microscope to visualise lignin auto-fluorescence and photographed using the Axiocam setup (Zeiss). Cloning, transformation and visualisation of GFP-tagged HvAP2 are described in the supplementary Materials and Methods.

### RNA extraction

All peduncle tissues were harvested from the main culm peduncle. For peduncle initial microarrays, a 1 mm tissue section below Bowman and *Zeo1.b* spike collars was collected from 25 individuals for each biological replicate (*n*=4) in the summer of 2014. From the same population and at one time point, 5 cm long Bowman peduncles and *Zeo1.b* 2 cm long peduncles were harvested and sectioned into 1 cm segments for the same time microarray. To compare peduncles at the same length, 2 cm long peduncles were harvested from Bowman and *Zeo1.b* plants grown in spring 2015 and sectioned into 1 cm segments; however, *Zeo1.b* tissue was 4 days older owing to the delay in peduncle elongation. Both 5 cm and 2 cm peduncle samples were harvested from five individuals per each biological replicate (*n*=4). Spikes were harvested from Bowman and *Zeo1.b* plants 3 weeks post germination. Spikes and unexpanded internodes were harvested from mock- and MeJA-treated plants 2 h following spray treatment (*n*=3). Tissues were flash-frozen and ground to a powder in liquid nitrogen before being re-suspended in 1 ml per 0.1 g tissue weight of TRI Reagent (Sigma), briefly vortexed and then spun at 12,000 ***g*** at 4°C for 10 min to pellet fibrous material. RNA extraction was carried out following manufacturer's recommendations with an additional chloroform extraction.

### Microarray, quality control and data extraction

RNA integrity was confirmed with a Bioanalyzer 2100 (Agilent Technologies). A custom Agilent gene expression microarray was used (Comadira et al., 2015). Microarrays were processed according to the ‘One-Color Microarray-Based Gene Expression Analysis’ protocol (v. 6.5; Agilent Technologies). Experimental design and complete datasets have been deposited in the ArrayExpress database at EMBL-EBI (www.ebi.ac.uk/arrayexpress) under accession numbers E-MTAB-7228, E-MTAB-7229, E-MTAB-7230, E-MTAB-7231. Data were extracted using Feature Extraction (FE) software (v. 10.7.3.1; Agilent Technologies) with default settings, and subsequently processed using GeneSpring GX (v. 7.3; Agilent Technologies) software. Data were normalised using Agilent FE one-colour settings: for each experiment, data were set to a minimum of 5 and normalised within each array to the 50th percentile of raw expression values, and individual probe data was subsequently normalised to its median value across all arrays. Flag-filtered data quality was visually assessed using box plots and performing Principal Component Analysis (PCA) for all replicates in each tissue sample using the R package ‘FactoMineR’ (Husson et al., 2016).

### Data filtering, clustering, GO enrichment and DEG analyses

Probes that were ‘Present’ or ‘Marginal’ in a minimum of three out of four biological replicates were considered to represent expressed genes. DEGs along the 1 cm segments of the 5 cm Bowman elongating peduncle were identified using ANOVA with a cut-off *P*-value of <0.05 whereas DEGs from *Zeo1.b* versus Bowman comparisons were identified using volcano filtering with a *t*-test *P*-value of <0.05. DEG lists from all comparisons were further filtered for two-fold change in expression between contrast groups. Hierarchical clustering was performed with the R function ‘hclust’ on the log2-transformed values of the 5 cm Bowman elongating peduncle and the dendrogram generated using the ‘ward.D’ function (Murtagh and Legendre, 2014; R Core Team, 2013) (https://www.R-project.org) to produce 25 co-expression clusters. Hierarchical clustering of the mean expression values of the clusters generated higher order megaclusters. To determine over-represented GO categories in DEG lists, custom GO identifiers for each DEG within a group along with the GO reference file (Fig. S19; see supplementary Materials and Methods for further details) were uploaded to the AgriGO website (http://bioinfo.cau.edu.cn/agriGO/analysis.php) for Singular Enrichment Analysis (Tian et al., 2017). The Hypergeometric test with a *P*-value cut-off of 0.05 along with the Bonferroni multiple testing correction calculated GO enrichment for each DEG list. Metabolic pathway reconstruction was carried out using the BarleyCyc 6.0 database and AP2-binding sites in selected DEGs were identified using PlantTFDB 4.0 (see supplementary Materials and Methods for further details).

### qRT-PCR

All qRT-PCR validation was performed using the same RNA analysed on the microarrays. Synthesis of cDNA was performed using the SuperScript VILO cDNA synthesis kit (Invitrogen) on 1 µg total RNA for each sample according to manufacturer's instructions. Synthesis of cDNA from mock- and MeJA-sprayed tissues was carried out with the Protoscript II kit (New England Biolabs) on 0.5 µg total RNA. To measure miR172, a separate cDNA synthesis reaction was conducted using a stem-loop miR172 primer (Chen et al., 2005). qPCR reactions were set up according to manufacturer's instructions (Taqman, Roche) using between 1:5 and 1:20 cDNA depending on the target amplicon, using primers (Table S4) designed from gene sequences derived from the full-length cDNA genes used to design the microarray (Comadira et al., 2015; IBSC, 2012) using the Universal ProbeLibrary (UPL) Assay Design Center (Roche) website. *JIP* and miR172 expression was measured using SYBR GREEN chemistry (Thermo Fisher). Each 48-well plate contained three biological replicates and three technical replicates. Endogenous controls used in this study were *ACTIN2* (*HvACT2*) and *PROTODERMAL FACTOR7* (*HvPDF7*), as in Houston et al. (2013). Reactions were run on the Applied Biosystems StepOne system.

### Hormone response assays

Gibberellic acid (GA_3_; Sigma) was dissolved in 95% ethanol to make a 100 mM stock solution. Plants were treated with either 0 M (mock), 0.001 mM or 0.01 mM GA_3_ in 95% ethanol. Droplets (20 μl) of each GA solution were applied every 4 days to the adaxial base of the youngest leaf of the main shoot beginning 14 dpg and continued for 4 weeks. Methyl jasmonate (Sigma) was dissolved in 95% ethanol to make a 100 mM stock solution. Experimental solutions were made by diluting the stock with water to 0 M, 1 mM or 5 mM MeJA with 0.5% Tween-20. Plants were sprayed with treatment solution and then loosely covered in a sealed bag for 2 h. This treatment continued every 2 days, starting at 14 dpg and continuing until 106 dpg. Leaf-segment unrolling tests were performed as in Dockter et al. (2014).

## Supplementary Material

Supplementary information
