# Supplementary material for: APETALA2 control of barley internode elongation
Source: Development. 2019 Jun 12;146(11):dev170373. doi: 10.1242/dev.170373 (PMC6589076; doi:10.1242/dev.170373)
Supplement: Supplementary information [file develop-146-170373-s1.pdf]

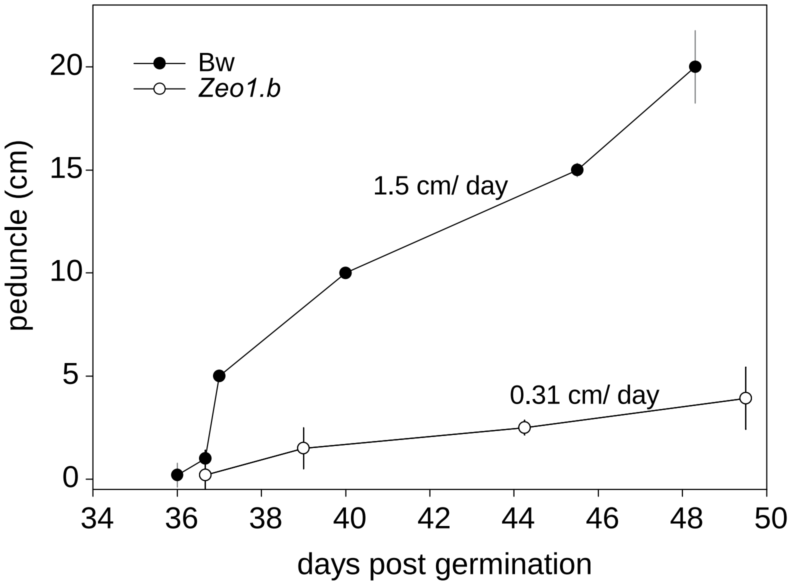

Fig. S1. Bowman and *Zeo1.b* peduncle growth. Peduncle length (cm) versus days post germination from onset of peduncle elongation until final length. Bw peduncles grew faster at 1.5 cm / day and to longer lengths than *Zeo1.b* which grew only 0.31 cm/ day. Error bars show s.e.m. (n = 3/ genotype).

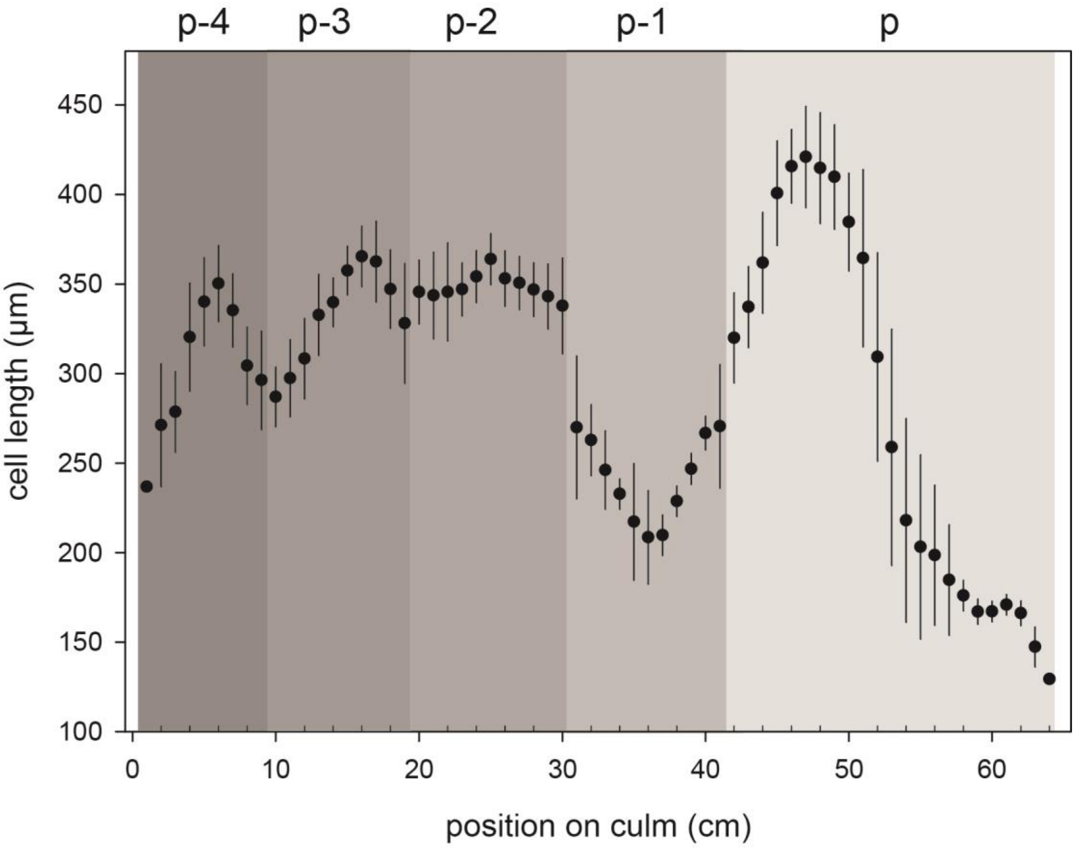

Fig. S2. Long cell length in centimetre segments along culm internodes in Bowman. Shaded regions correspond to individual internodes labelled above with respect to the peduncle internode (p). Only the peduncle showed acropetal gradient of very long to short long cells. Error bars show s.e.m. (n = 3).

Development • Supplementary information

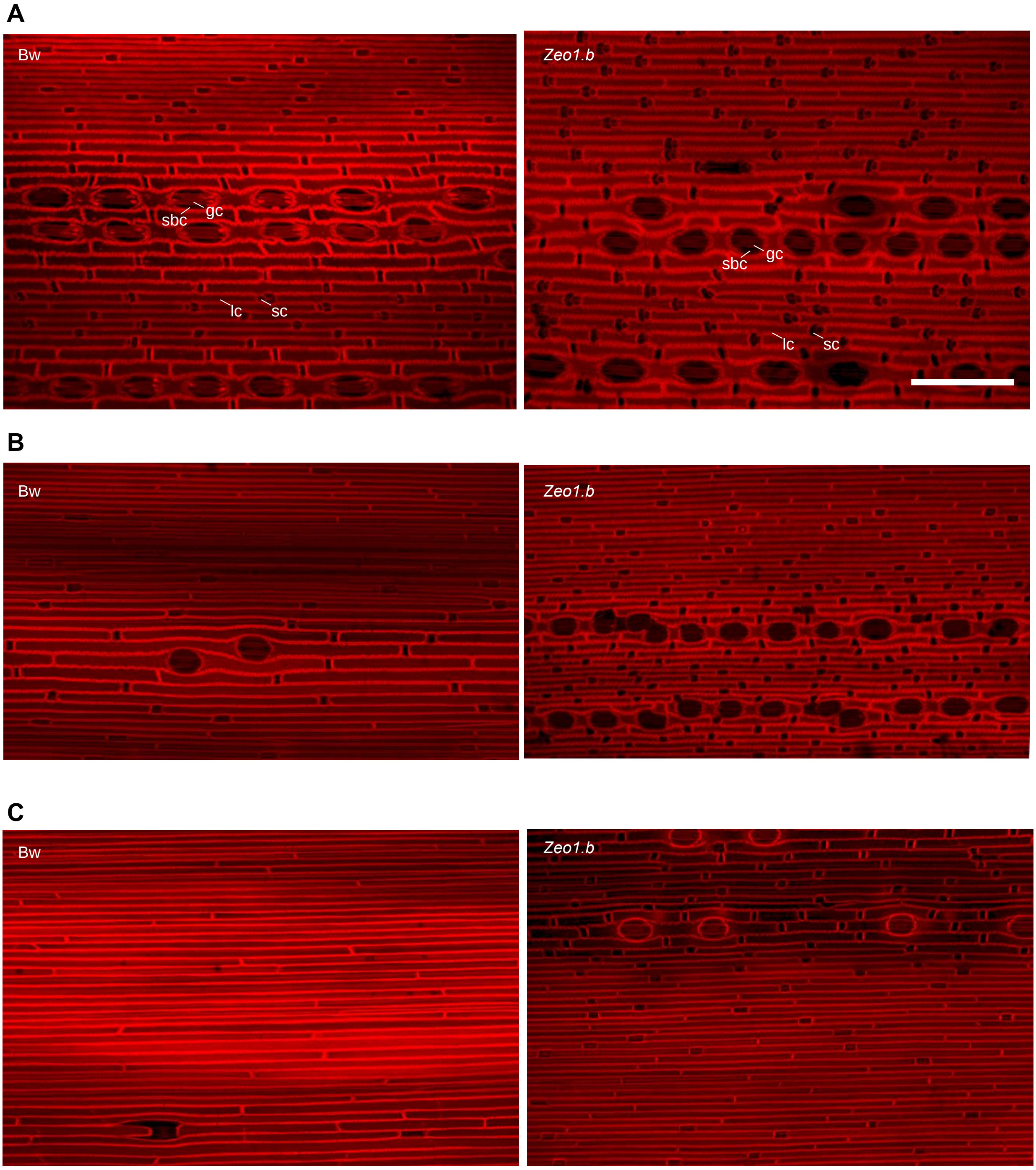

Fig. S3. Epidermis of Bowman and *Zeo1.b* peduncles. Propidium iodide-stained epidermis from apical (A), middle (B) and basal (C) regions of Bowman (left) and *Zeo1.b* (right) peduncles. Scale in (A) applies to all panels, 100 µm. gc, guard cell; lc, long cell; sbc, subsidiary cell; sc, silica-cork cell.

Development • Supplementary information

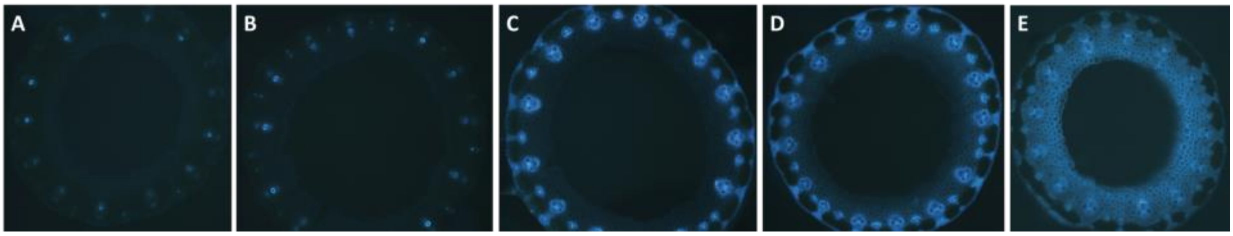

Fig. S4. Lignification profile of the 5 cm Bowman peduncle. 5 cm peduncles were segmented into five equal 1 cm segments. Panels (A-E) show lignin autofluorescence from peduncle cross-sections from the middle of each segment, arranged from bottom to top of the peduncle. (n = 3).

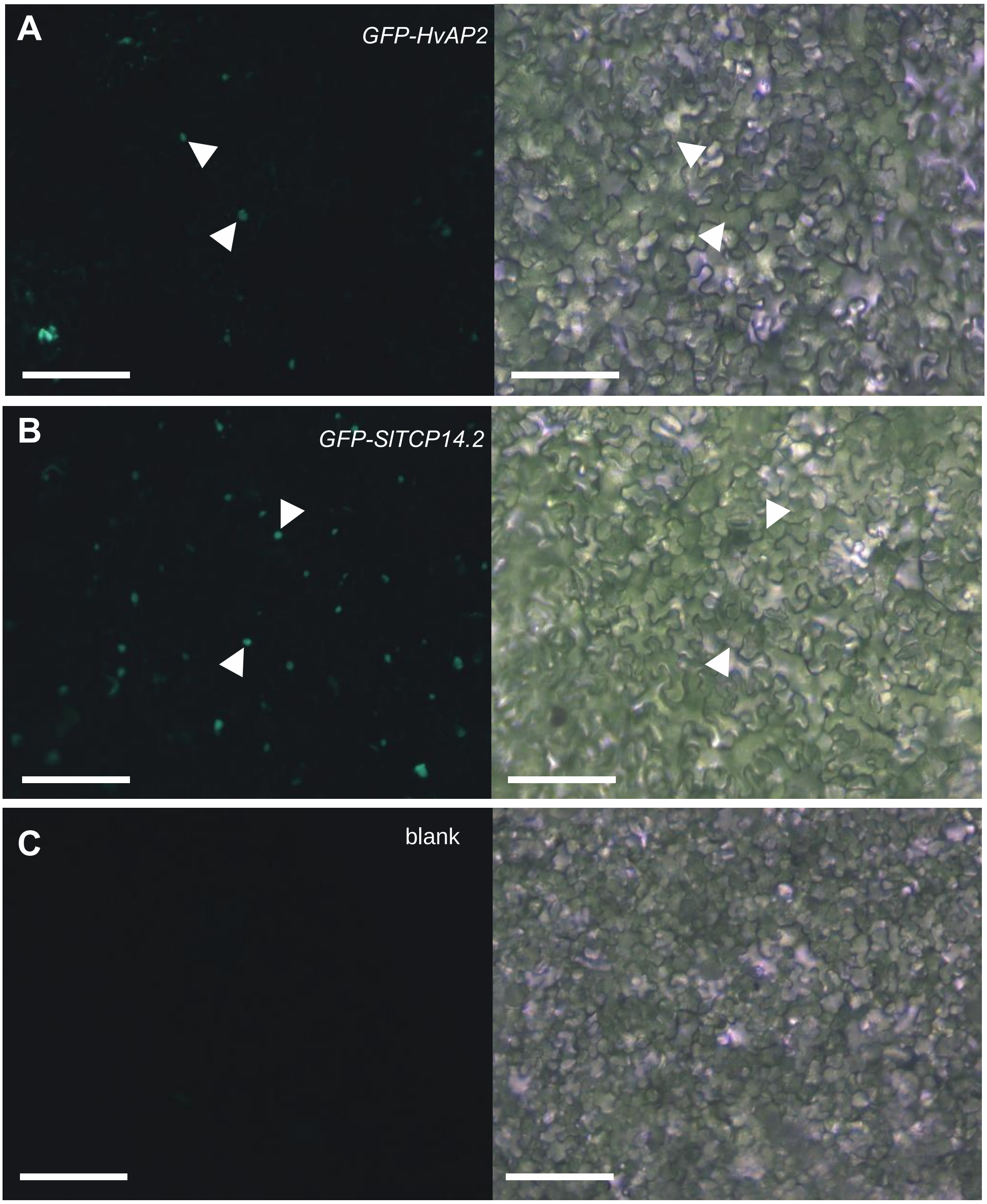

Fig. S5. Nuclear localisation of HvAP2. (A-C) Fluorescence (left panels) and brightfield (right panels) images of transiently transformed *Nicotiana benthamiana* abaxial leaf sections. (A) Leaf containing *GFP-HvAP2* showing nuclear localisation. (B) Leaf infiltrated with Agrobaterium expressing a tomato nuclear-localised TCP transcription factor, GFP-SlTCP14.2, as a positive control for nuclear localisation. (C) Control leaf infiltrated with Agrobacterium with no construct (blank). White arrowheads indicate fluorescent nuclei with location also shown in brightfield. Scale bars, 2 mm.

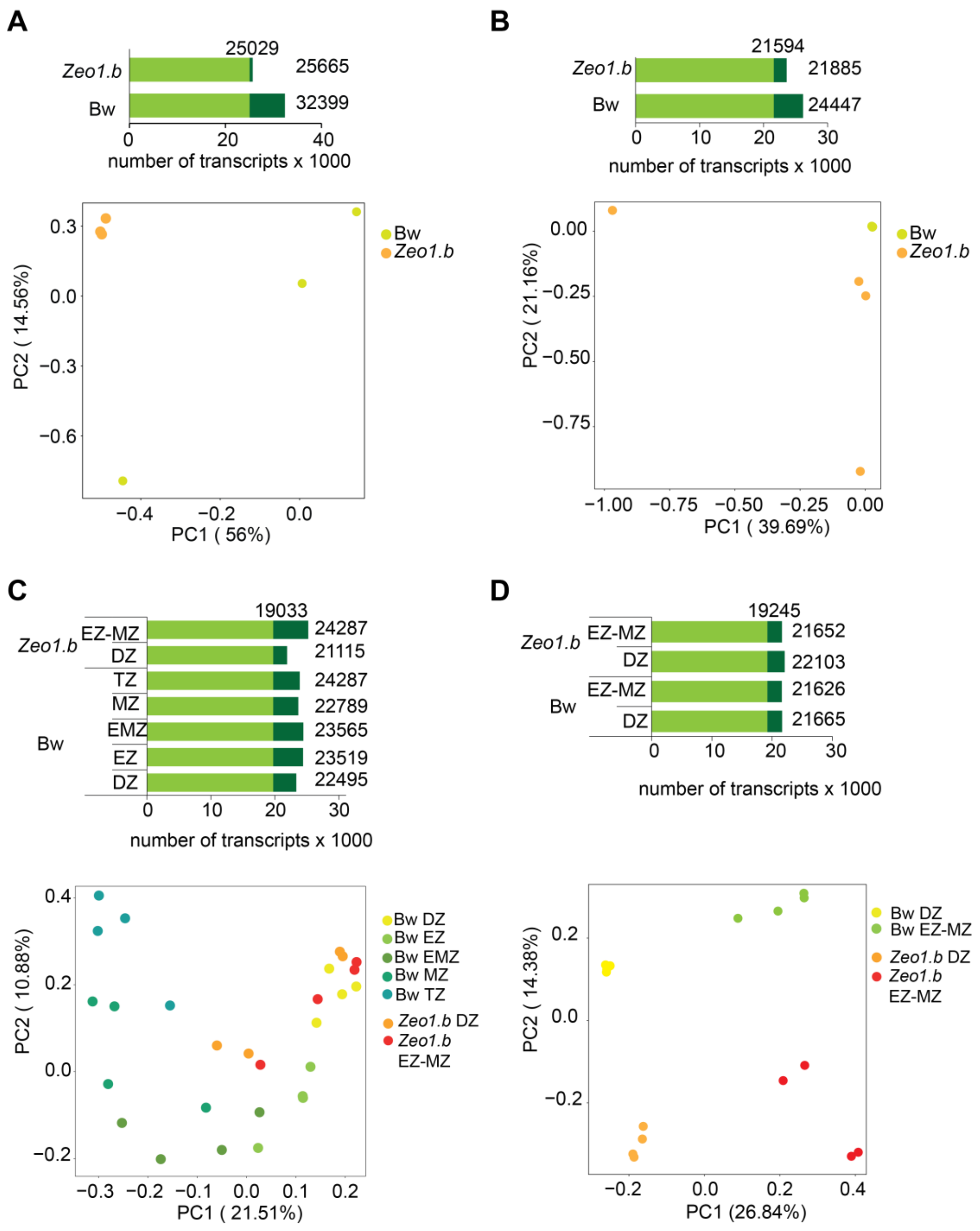

Fig. S6. Microarray Quality control. (A-D) Probes detected (top) and PCA of bioreplicates (bottom). Light green section denotes shared probes and dark green segments denote unique probes. Dots represent individual bioreplicates. (A) Microarray comparing Bowman and *Zeo1.b* spikes at awn primordium stage. (B) Microarray comparing Bowman and *Zeo1.b* peduncle initials. (C) Microarray comparing 5 cm Bowman peduncles (segmented into zones) with 2 cm *Zeo1.b* peduncles (segmented into 1 cm sections) in the same time microarray. (D) Microarray comparing Bowman and *Zeo1.b* 2 cm peduncles (segmented into 1 cm sections) in the same length microarray. DZ, division zone; EZ, expansion zone; EMZ, expansion-maturation transition zone; MZ, maturation zone; TZ, termination zone.

Development • Supplementary information

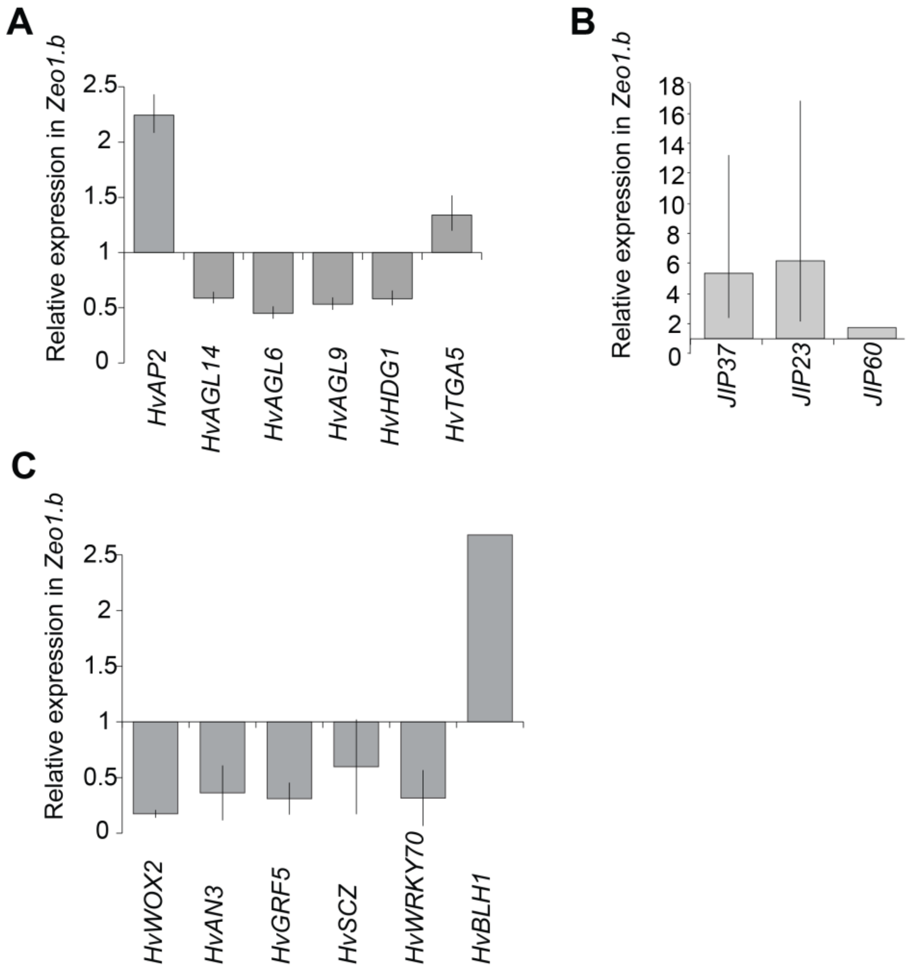

Fig. S7. Validation of differentially expressed genes (DEGs) between Bowman and *Zeo1.b*. Expression of DEGs were assessed by qPCR and plotted as the *Zeo1.b* expression relative to Bowman. (A) DEGs between Bowman and *Zeo1.b* peduncle initials. (B) DEGs between Bowman and *Zeo1.b* spikes. (C) DEGs between Bowman and *Zeo1.b* peduncle DZs. *WOX2*, *WUSHCEL-LIKE HOMEOBOX2*; *AN3*, *ANGUSTIFOLIA3*; *GRF5*. *GROWTH REGULATORY FACTOR4*; *SCZ*, *SCHIZORIZA*; *BLH1*, *BEL1-LIKE HOMEODOMAIN*; *AP2*, *APETALA2*; *AGL14*, *AGAMOUS-LIKE14*; *AGL6, AGAMOUS-LIKE6*; *AGL9*, *AGAMOUS-LIKE9*; *HDG1*, *GLABRA-LIKE1 HOMEODOMAIN*; *JIP*, *JASMONATE INDUCED PROTEIN*. (n = 3/ genotype). Error bars show s.e.m.

Development • Supplementary information

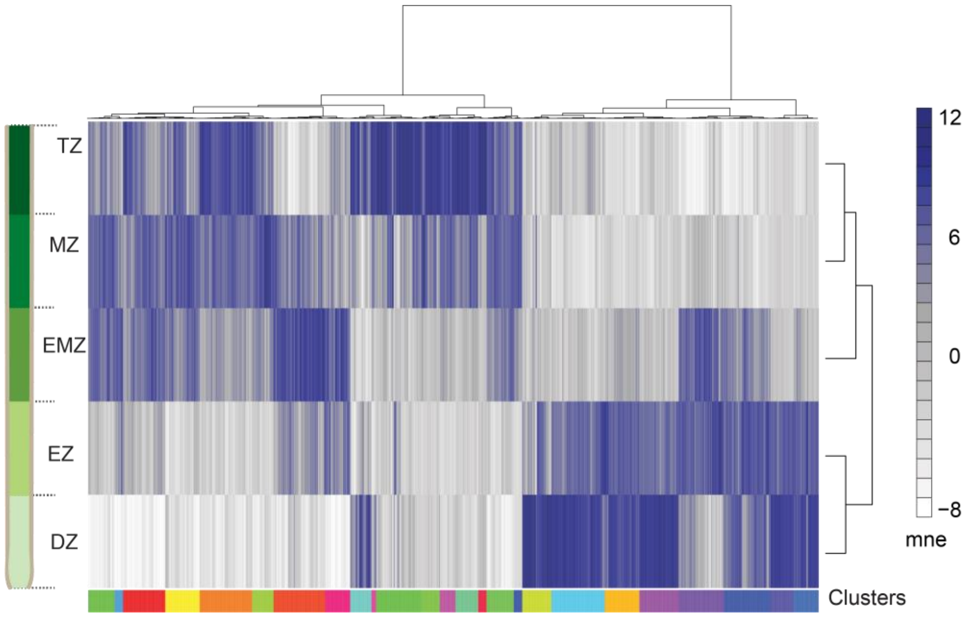

Fig. S8 Hierarchial clustering of the elongating peduncle transcriptome. Heat map of differentially expressed genes generated following hierarchial clustering, Co-expression clusters are arranged in colour blocks underneath the heat map. Scale shown on right. mne, mean normalised expression. DZ, division zone; EZ, expansion zone; EMZ, expansion-maturation transition zone; MZ, maturation zone; TZ, termination zone

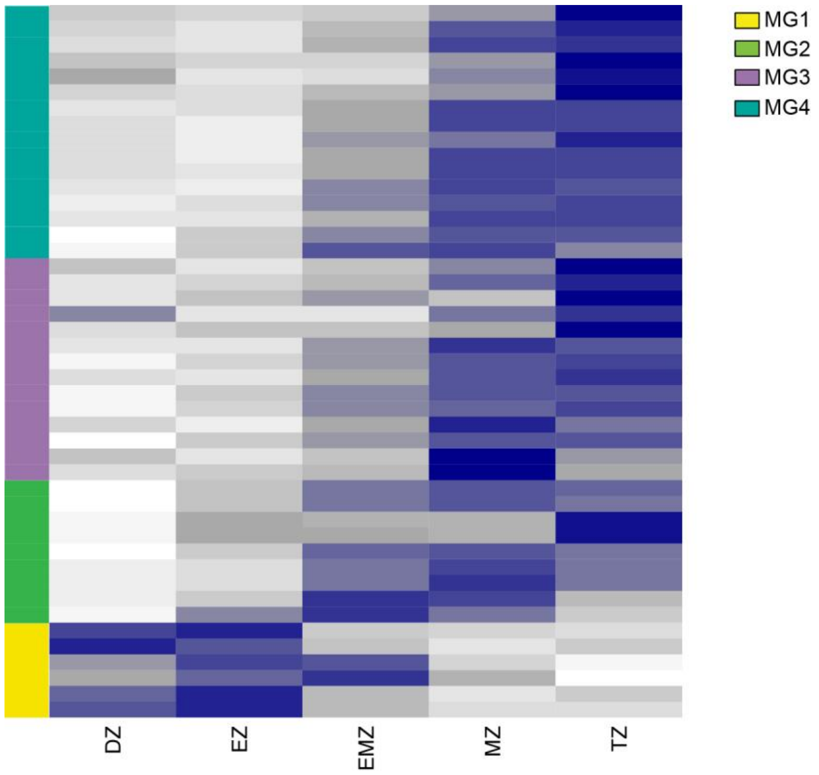

Fig. S9. Heat map of differentially expressed genes (DEGs) annotated for phenylpropanoid metabolism. Association of each DEG with its megacluster (MG) is shown on the bar to the left**.** DZ, division zone; EZ, expansion zone; EMZ, expansion-maturation transition zone; MZ, maturation zone; TZ, termination zone.

Development • Supplementary information

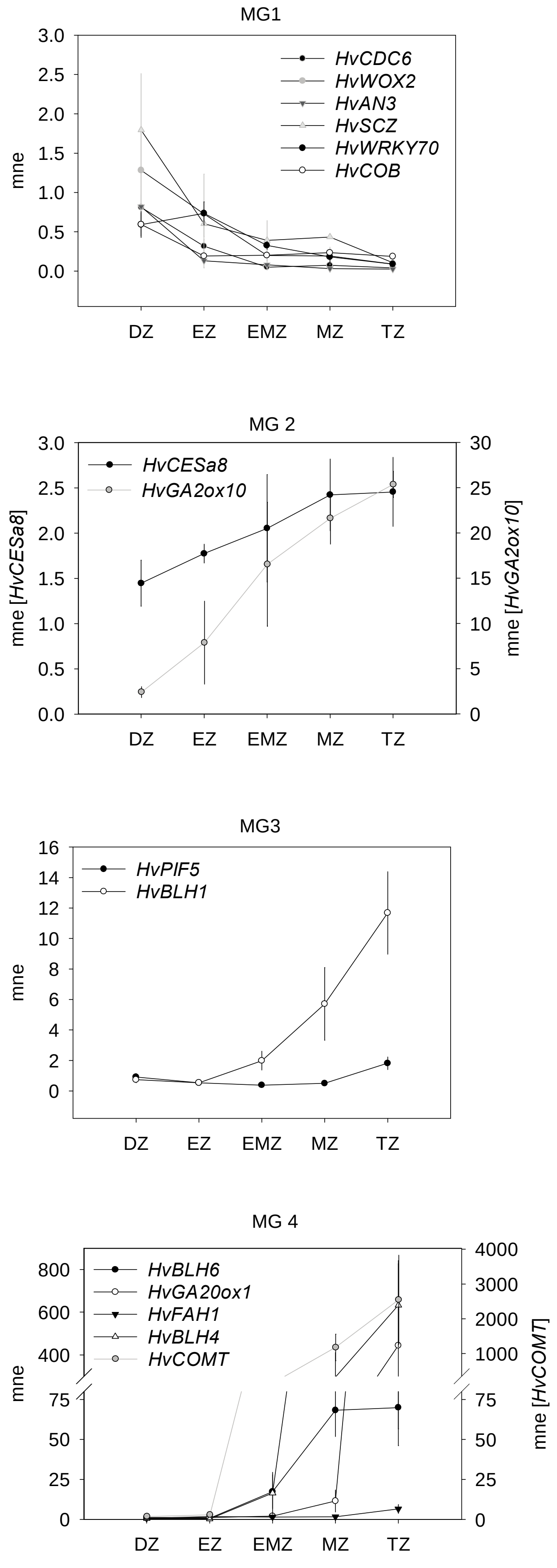

Fig. S10. Validation of differentially expressed genes (DEGs) along the elongating peduncle. Expression of DEGs from each megacluster (MG) within each peduncle zone were assessed by qPCR. DZ, division zone; EZ, expansion zone; EMZ, expansion-maturation transition zone; MZ, maturation zone; TZ, termination zone. mne, mean normalised expression. Error bars show s.e.m. (n = 3/ developmental zone).

Development • Supplementary information

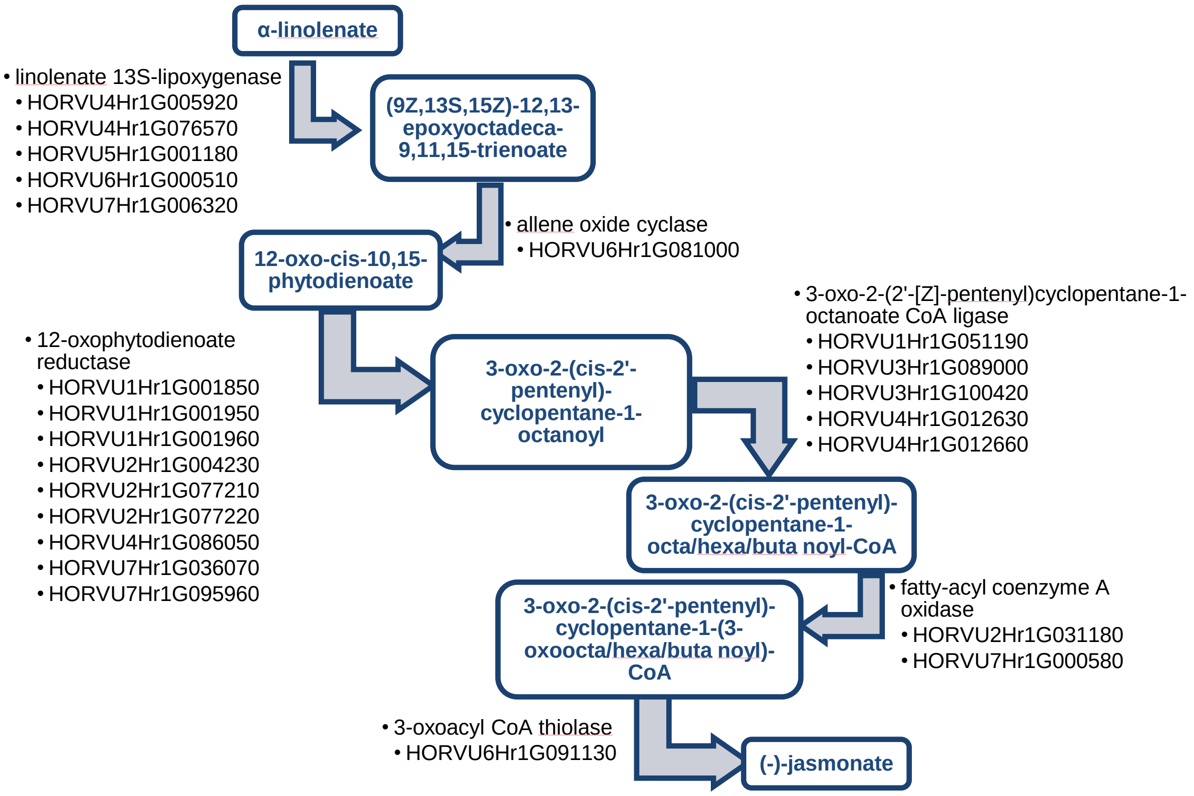

Fig. S11 Jasmonate biosynthesis pathway with barley gene models. Simplified JA biosynthesis pathway showing key steps and associated enzymes. Encoding barley HORVU gene models which contain AP2 binding motifs in their promoters are shown.

Development • Supplementary information

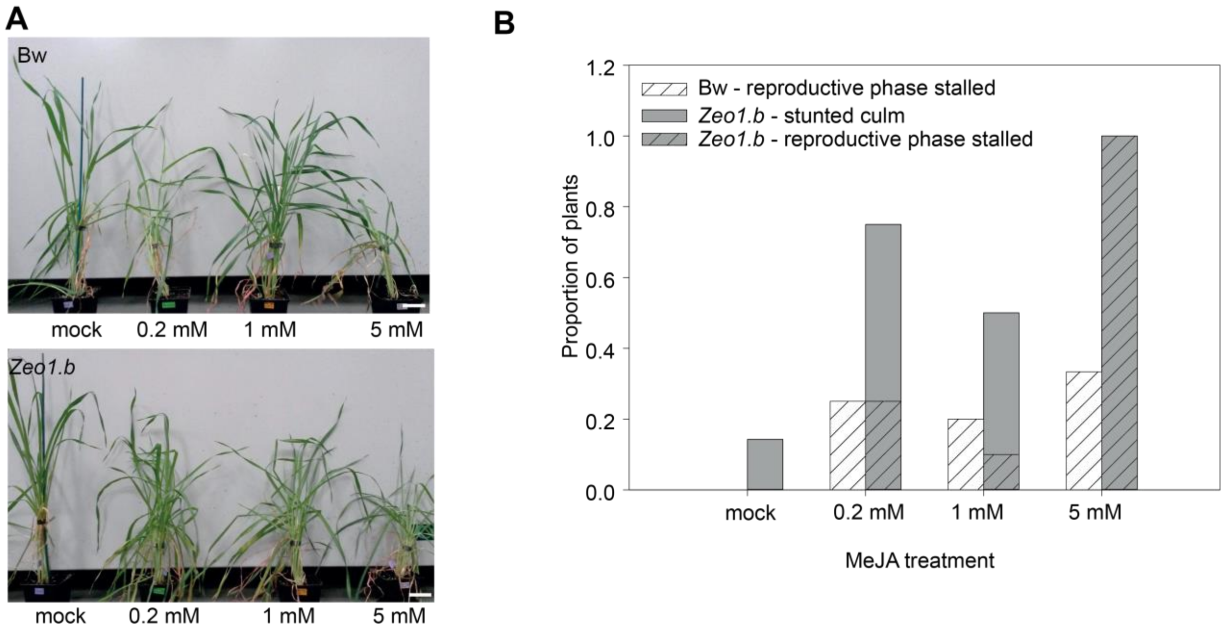

Fig. S12 Application of MeJA stalls reproductive development. (A) Photos of entire plants treated with either mock or 0.2 mM and 1 mM MeJA. (B) Proportion of plants with either stunted culms or stalled reproductive development following four weeks treatment. MeJA led to variable stalling of reproductive development, while a larger proportion of Bowman plants lacked a mature spike or elongated stem when treated with 5 mM MeJA; in contrast, all *Zeo1.b* plants treated with either 1 mM or 5 mM MeJA lacked stem elongation altogether and none of the 5mM-treated spikes developed beyond green anther stage. MeJA was suspended in 95% ethanol in this trial.

Development • Supplementary information

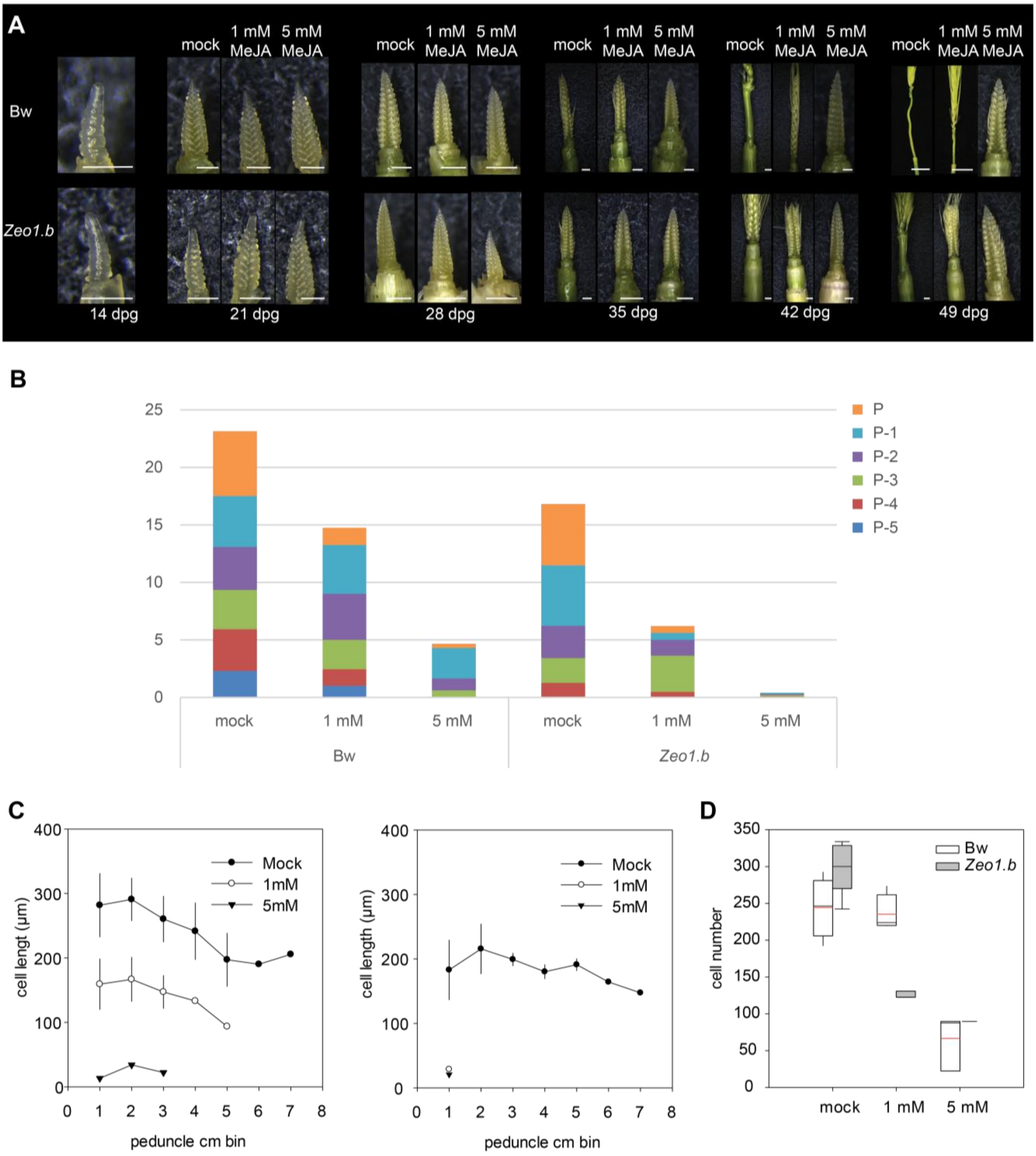

Fig. S13 Developmental effects of MeJA application on Bowman and *Zeo1.b* plants grown in growth cabinets. (A) Apex development during MeJA application. Plants were sprayed with either mock, 1mM or 5mM MeJA in 0.05% Tween-20. Pictures of representative shoot apices of Bowman and Zeo1.b (n ≥ 3/ genotype/ time point) at the start of MeJA treatment (14 days post-germiantion, dpg) and at subsequent time points. (B) Final internode lengths measured at 106 dpg. (n=4-6/ genotype/ treatment). (C) Cell length of long cells in each 1 cm peduncle segment of mock, 1mM and 5mM MeJA-treated Bowman (right panel) and *Zeo1.b* (left panel), (n=3-5/ genotype/ treatment). (D) Long cell numbers per file in mock, 1mM and 5mM MeJA-treated Bowman and *Zeo1.b* peduncles. (n=3-5/ genotype/ treatment). Box plots show 25th to 75th percentiles; whiskers extend down to 10th and up to 90th percentiles; black line shows median; and red line shows mean. Scale bars: 1 mm (except Bw dpg 49 mock and 1mM MeJA-treated, 1 cm).

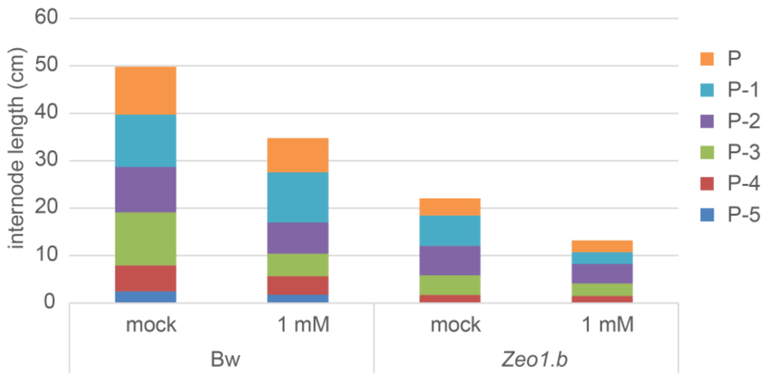

Fig. S14 MeJA application of glasshouse-grown plants. Final internode lengths measured at 100 days post germination. (n = 10-14/ genotype/ treatment)

Development • Supplementary information

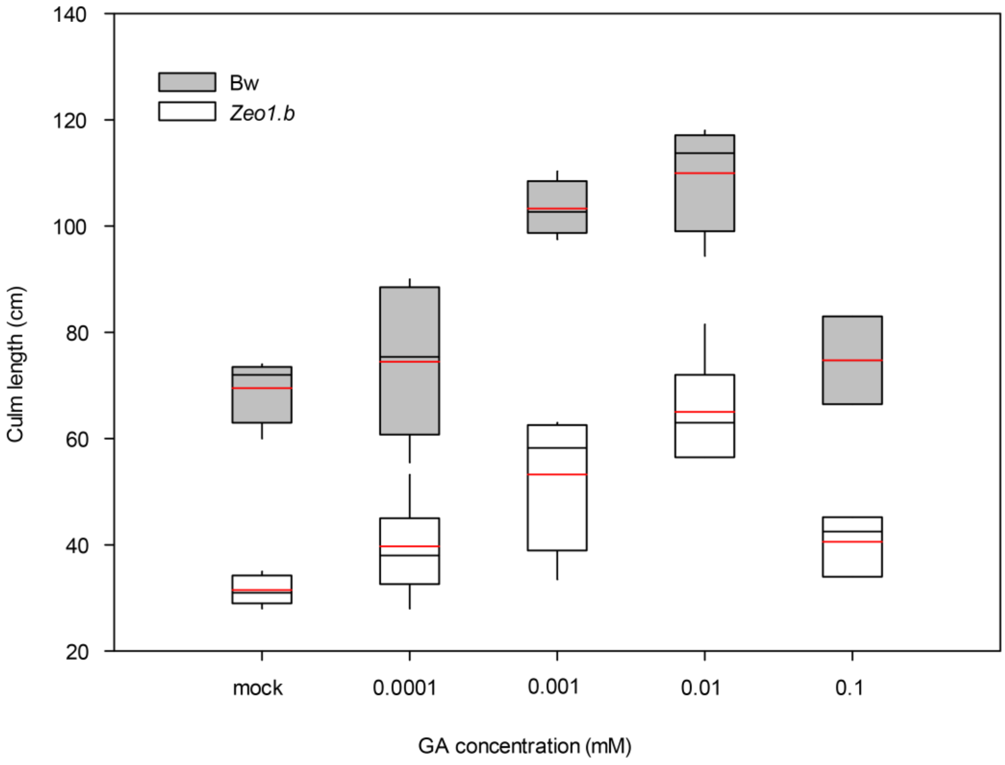

Fig. S15 Dose-response trial experiment of gibberellin (GA_3) application on Bowman and *Zeo1.b*. Mature culm lengths of glasshouse-grown plants were treated with droplets of GA every four days starting 14 days post-germination (dpg). (n = 4-10/ genotype/ treatment). Box plots show 25th to 75th percentiles; whiskers extend down to 10th and up to 90th percentiles; black line shows median; and red line shows mean.

Development • Supplementary information

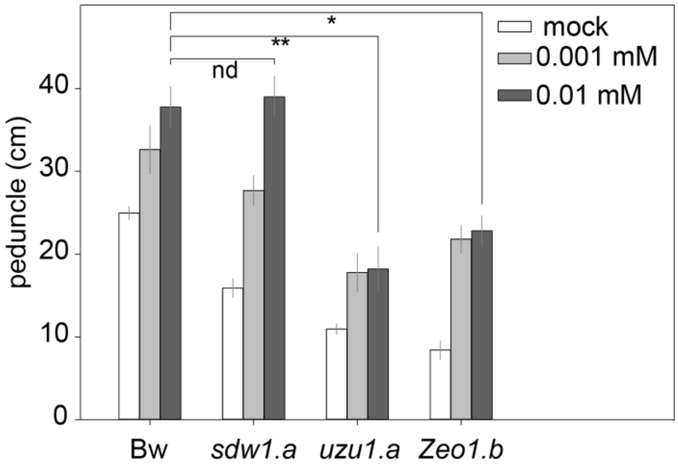

Fig. S16. Peduncle length in response to GA_3 treatment in Bowman and semi-dwarf mutants. Peduncle lengths of Bowman, *sdw1.a*, *uzu1.a* and *Zeo1.b* plants following treatment with mock, 0.001 mM and 0.01 mM GA_3. (n = 7-10/ genotype/ treatment). Bw, Bowman. **P*<0.01; ***P*<0.05 (Student's t-test).

Development • Supplementary information

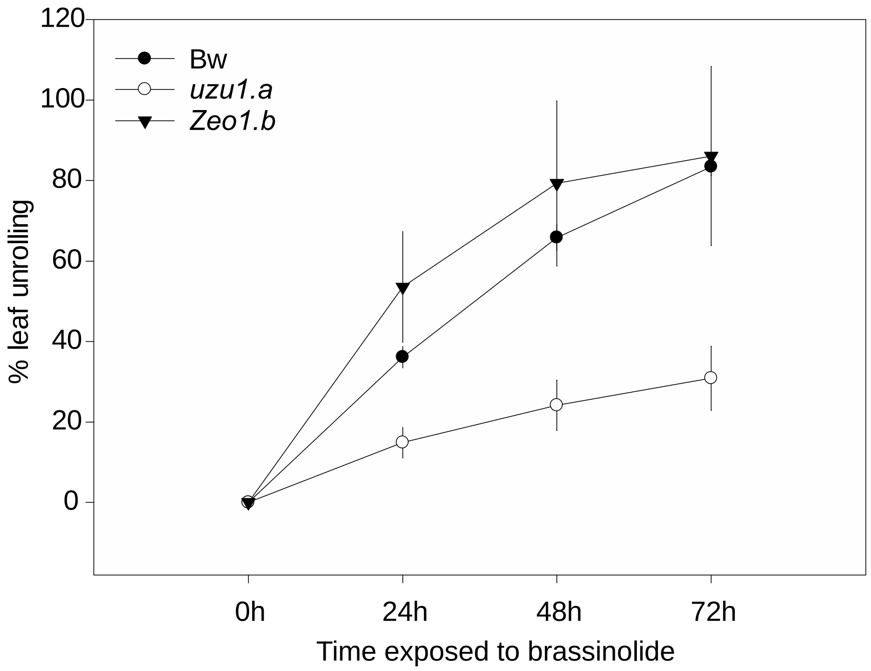

Fig. S17. Leaf-segment unrolling assays in Bowman, *uzu1.a* and *Zeo1.b*. Bowman and *Zeo1.b* leaves show rapid unrolling upon exposure to exogenous brassinolide while *uzu1.a* shows insensitivity. h, hours. Error bars show s.e.m. (n = 15/ genotype)

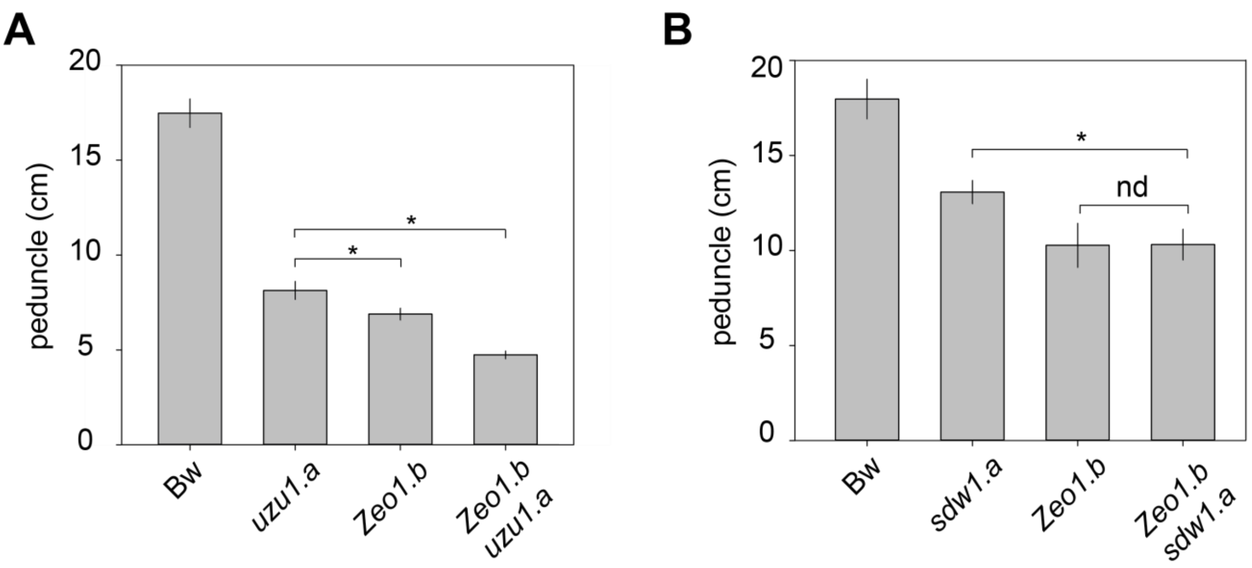

Fig. S18. Peduncle lengths of *Zeo1.b sdw1.a* and *Zeo1.b uzu1.a* compared to parents and Bowman. (A) Peduncle lengths of Bowman, *uzu1.a*, *Zeo1.b* and *uzu1.a Zeo1.b* mutants. (n= 7-19/ genotype). (B) (F) Peduncle lengths of Bowman, *sdw1.a*, *Zeo1.b* and *sdw1.a Zeo1.b* mutants. (n = 10/ genotype).
Error bars show s.e.m.. **P*<0.05 (Student's t-test). Bw, Bowman.

Supplementary Materials and Methods

Cloning, transformation and visulisation of GFP-HvAP2

The HvAP2 CDS was cloned into pENTR1A flanked by recombination sites (attL and attR). HvAP2 was fused to GFP by Gateway mediated recombination into the destination vector pK7WGF2. The GFP-HvAP2 construct was confirmed by sequencing and transformed into *Agrobacterium tumefaciens* (*Agrobacterium*) strain GV3101 by electroporation. Colony PCR confirmed the transformation of GFP-HvAP2 in independent colonies.

Nicotiana benthamiana (*N.benth*) were grown in a greenhouse at 22-25 °C with 16 h photoperiod. *Agrobacterium* containing GFP-HvAP2 as well as a positive control line containing EGFP-STICP 14.2 (Stam et al., 2013), and a negative control line without vector were infiltrated into *N. benth* leaves. Prior to infiltration, *Agrobacterium* lines were grown overnight in liquid broth culture, pelleted and then diluted in infiltration buffer (OD = 0.1) before syringe infiltration into *N.benth* leaves. Approximately 0.25 ml of *Agrobacterium* suspension was injected into four points on each abaxial leaf surface. After 48 hours further growing time, leaves were imaged with a 450-490 nm excitation filter over a Mercury-vapor lamp. Bright field images were also acquired for the same location as fluorescence images.

Assigning GO terms to sequences from the barley 61k chip

Peptide sequences for *Arabidopsis* were obtained from The *Arabidopsis* Information Resource (TAIR) at https://www.arabidopsis.org/ (Lamesch, *et al*., 2012) and peptide sequences for rice were obtained from the Rice Annotation Project Database (RAP-DB) at http://rapdb.dna.affrc.go.jp/ (Sakai, *et al*., 2013) and from the Michigan State University (MSU) Rice Genome Annotation Project database at http://rice.plantbiology.msu.edu/ (Kawahara, *et al*., 2013). Both sources were chosen for rice as later mapping of identifiers to Gene Ontology (GO) terms using g:Profiler (Reimand, *et al*., 2016) required RAP IDs rather than MSU IDs.

BLASTX searching of the barley sequences against the rice and *Arabidopsis* peptides was carried out to identify top-ranked hits for each, but with otherwise default parameters (E=10) to allow for downstream filtering of results. Matches were filtered based on percentage identity over percentage of the query (barley) sequence aligned: 50% identity over 50% query sequence for rice matches and 40% identity over 50% query sequence for *Arabidopsis* matches. In cases where no RAP-DB match was identified using BLAST but an MSU match was identified, then those MSU IDs could be directly converted into RAP-IDs using the RAP-DB ID converter. RAP-DB IDs for those mapping unambiguously were then included in the set. Further RAP-DB and TAIR matches were also obtained as part of a pilot GO Slim analysis carried out at the start of this study. Whilst many of these did not pass the criteria of percentage identity / percentage query length, those matches that had BLAST E-value scores of 1e-5 or lower were also added to the sets for g:Profiler analysis.

Accession numbers from RAP-DB and TAIR were then supplied to g:Profiler at http://biit.cs.ut.ee/gprofiler/ (Reimand, *et al*., 2016) to identify the sets of GO terms associated with each. Default parameters were used, but the option to return only significant terms was deselected as this would cause g:Profiler to attempt an enrichment analysis, but at this stage of the analysis all matching terms were required.

GO terms arising from the RAP-DB ID mapping were chosen in preference to those arising from TAIR ID mapping since barley is more closely related to rice than it is to *Arabidopsis*, meaning that in situations where GO terms were returned for a barley sequence from both RAP-DB and TAIR mappings, no attempt was made to merge the two lists so as to avoid possible conflicting terms. Mappings arising from TAIR would only be used where there was no corresponding information from RAP-DB.

The resulting set of GO terms can then be used for downstream enrichment analysis.

Of the 61,487 barley sequences 58,055 matched to *Arabidopsis* peptides from the TAIR set (E=10), with 24,639 matching with at least 40% identity over at least 50% of the query sequence length; 57,666 matched to rice peptides from the MSU set (E=10) and, of these 29,798 matched with at least 50% identity over at least 50% of the query (barley) sequence length; from the RAP-DB set 58,716 sequences were matched (E=10) with 27,798 matching with at least 50% identity over 50% query sequence length. Additional direct mapping of MSU IDs and inclusion of matches from the pilot GO Slim analysis resulted in final sets of 31,134 barley sequences with corresponding RAP-DB IDs and 26,039 barley sequences with corresponding TAIR IDs. These were then supplied to g:Profiler to identify the GO terms associated with each.

Development • Supplementary information

Not all RAP-DB or TAIR IDs returned lists of GO terms. GO terms arising from RAP-DB mappings were assigned to 24,077 barley sequences and terms arising from TAIR mappings were assigned to 24,295 barley sequences. Merging these lists produced a final set of 29,787 barley sequences with 8,222 associated GO terms arising from either RAP-DB or TAIR mappings and with a maximum term depth level of 15. Figure S19, generated using Venny (Oliveros, 2007-2015) shows the distribution of barley genes with GO terms arising from RAP-DB or TAIR mappings.

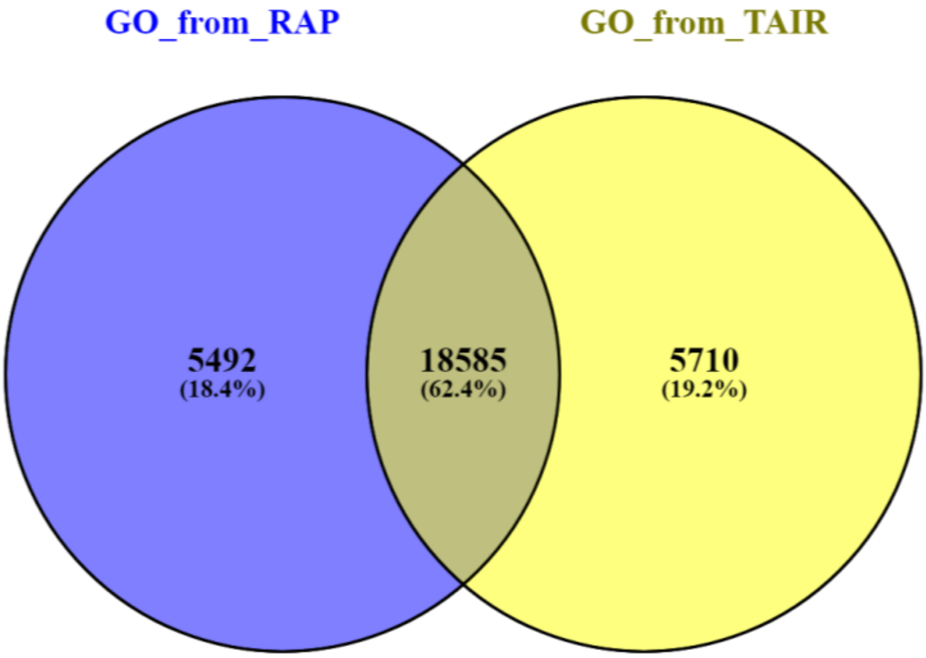

Fig S19. Number of genes with GO term mappings arising from either RAP-DB or TAIR

Metabolic Pathway Reconstruction along the internode

To relate the probe sequences of the barley microarray to the latest HORVU gene models, the probes were aligned to the full set of HORVU transcripts sequences using the blastn command line tool (version blast+ 2.28, Camacho et al. 2009, Altschul et al. 1990). The output from this was filtered to retain only those hits with 100% query coverage and an identity value of >=95%, leaving a single HSP per query. Duplicate combinations of query x gene were then removed to eliminate multiple transcripts from the same gene that had been hit by a given query. Of the 61487 microarray probes, 42466 were associated with a HORVU transcript ID. The 36995 unique HORVU transcript IDs had 30479 unique HORVU gene IDs, which were used for the analysis of the hormone pathway genes. BarleyCyc 6.0 database (https://www.plantcyc.org/databases/barleycyc/6.0) was used to identify genes encoding enzymes from the gibberellin, brassinosteroid and jasmonic acid pathways along with their corresponding HORVu accession number. These HORVus were then filtered for those expressed in the Bowman peduncle.

Identification of potential APETALA2 binding sites

The 500bp promoters of differentially expressed genes in *Zeo1.b* compared to Bowman or metabolic pathway genes were retrieved from Ensembl (Frankish et al. 2017) Plants Genes 42, Hordeum vulgare genes (IBSC v2) using the Biomart tool (https://plants.ensembl.org/biomart/martview/a4076bb018d3718a542b11cf7d46d092) via HORVu accession number. Where multiple barley accessions corresponded to HORVu accessions, all possible barley accessions were considered. These sequences were analysed using the PlantTFDB 4.0 (Jin et al. 2017) for potential binding sites of known barley transcription factors Potential APETALA2 binding sites were identified in these sequences using the Find Individual Motif Occurrences within the MEME Suite 5.2 using a false discovery cut off of q > 0.05. These motifs were then filtered for those containing consensus AP2-binding motif 'AACAAA' or 'TTTGTT' identified in Dinh et al (2012).

References

Altschul, S. F., et al. (1990). "Basic local alignment search tool." *Journal Molecular Biology* 215(3): 403-410.

Camacho, C., et al. (2009). “BLAST+: architecture and applications." *BMC Bioinformatics* 10: 421.

Dinh, T. T., T. Girke, X. Liu, L. Yant, M. Schmid and X. Chen (2012). "The floral homeotic protein APETALA2 recognizes and acts through an AT-rich sequence element." *Development* **139**(11): 1978-1986.

Frankish, A., A. Vullo, A. Zadissa, A. Yates, A. Thormann, A. Parker, A. Gall, B. Moore, B. Walts, B. L. Aken, et al (2017). "Ensembl 2018." *Nucleic Acids Research* **46**(D1): D754-D761.

Jin, J., F. Tian, D. C. Yang, Y. Q. Meng, L. Kong, J. Luo and G. Gao (2017). "c" *Nucleic Acids Research* **45**(D1): D1040-d1045.

Kawahara, Y.; de la Bastide, M.; Hamilton, J.P.; Kanamori, H.; McCombie, W.R.; Ouyang, S.; Schwartz, D.C.; Tanaka, T.; Wu, J.; Zhou, S.; Childs, K.L.; Davidson, R.M.; Lin, H.; Quesada-Ocampo, L.; Vaillancourt, B.; Sakai, H.; Lee, S.S.; Kim, J.; Numa, H.; Itoh, T.; Buell, C.R.; Matsumoto, T. (2013): Improvement of the *Oryza sativa* Nipponbare reference genome using next generation sequence and optical map data. *Rice* **6:** 4

Lamesch, P.; Berardini, T.Z.; Li, D.; Swarbreck, D.; Wilks, C.; Sasidharan, R.; Muller, R.; Dreher, K.; Alexander, D.L.; Garcia-Hernandez, M.; Karthikeyan, A.S.; Lee, C.H.; Nelson, W.D.; Ploetz, L.; Singh, S.; Wensel, A.; Huala, E. (2012): The *Arabidopsis* Information Resource (TAIR): improved gene annotation and new tools. *Nucleic Acids Research* **40** (Database Issue): D1202-D1210

Oliveros, J.C. (2007-2015) Venny. An interactive tool for comparing lists with Venn's diagrams. http://bioinfogp.cnb.csic.es/tools/venny/index.html

Reimand, J.; Arak, T.; Adler, P.; Kolberg, L.; Reisberg, S.; Peterson, H.; Vilo, J. (2016): g:Profiler – a web server for functional interpretation of gene lists (2016 update). *Nucleic Acids Research* **44** (W1): W83-W89

Sakai, H.; Lee, S.S.; Tanaka, T.; Numa, H.; Kim, J.; Kawahara, Y.; Wakimoto, H.; Yang, C.C.; Iwamoto, M.; Abe, T.; Yamada, Y.; Muto, A.; Inokuchi, H.; Ikemura, T.; Matsumoto, T.; Sasaki, T.; Itoh, T. (2013): Rice Annotation Project Database (RAP-DB): an integrative and interactive database for rice genomics. *Plant Cell & Physiology* **54** (2): e6(1-11)

Stam et al (2013) conserved oomycete CRN effector targets and modulates tomato TCP14-2 to enhance virulence https://www.biorxiv.org/content/early/2013/12/11/001248

Development • Supplementary information

Table S1. Comparative gene expression in *Zeo1.b* versus Bowman

Click here to Download Table S1

Table S2. Gene ontogeny enrichment

Click here to Download Table S2

Table S3. Promoter motif analyses

Click here to Download Table S3

Development • Supplementary information

Table S4. Primers used for *in situ* hybridisation and qRT-PCR

qPCR

Gene Model	Primer name_UP	Sequence 5'...3'	R^2	Efficien
MLOC_7641	AGL14_UP70_L<br>AGL14_UP70_R	CTCTATCCGCCGCAACTC<br>GAGGATGAGCATTTGAAGACG	0.97	109.31
AK374424	AGL9_UP32_L<br>AGL9_UP32_R	AGGAGAGCAGCCAGCAGAT<br>CGTAGCCGAGCAAGTTGG	0.99	103.05
AK365794	HDG1_UP2_L<br>HDG1_UP2_R	ACCCAGATGAAGACGCAGAT<br>CCCGGATCGTCATGTTCT	0.99	104.75
AK365938	COB_UP15_L<br>COB_UP15_R	GGCATCGTTTCAGATCACTG<br>TCGGTGCTTTCACTGTCCTA	0.99	99.68
MLOC_1761	TGA5_UP70_L<br>TGA5_UP70_R	CCATTTCTCCGACTGATCTAGC<br>GTGCCTGAGGATCAACTGCT	0.99	101.88
MLOC_1543	WRKY70_UP149_<br>WRKY70_UP149_	AGACGAGGCCGAGAAGAAG<br>CTCCATATGAACCCGTCCTC	0.99	102.58
MLOC_5369	WOX3_UP75_L<br>WOX3_UP75_R	CGTGTCCTCCACAACTTGG<br>CGTAGGATCGCCGGAATAC	0.97	105.93
MLOC_7733	PIF5_UP142_L<br>PIF5_UP142_R	CCAGATGCAAAACAATGGTG<br>ATGCATAGGGCAACATACCC	0.98	98.91
AK356936	EXP1_UP142_L<br>EXP1_UP142_R	GTGCCGGTCCTCTACCAG<br>GTTGACGGTGAACCTGACG	0.99	107.65
AK353813	GRF5_UP127_L<br>GRF5_UP127_R	GTTACCACCACGGTGAATGA<br>GCAGCAATCCAACACTTCG	0.99	102.92
AK375249	AN3_UP77_L<br>AN3_UP77_R	GATGTAGCGTCGGATGTCG<br>GCACTACATAGGGAGTGTTCACAA	0.98	102.03
MLOC_7335	SCZ_UP7_L<br>SCZ_UP7_R	CCGCCAGCTCAACACCTA<br>GTGGATCTCGCACAGCAG	0.99	102.32
MLOC_6288	WOX2_UP46_L<br>WOX2_UP46_R	CTGTGAGTAGTAGGTTGGATTAGCTG<br>CAACGGAGAAGCAACGTACA	0.99	103.35
AK251179.1	DNALigaseIV_UP<br>DNALigaseIV_UP	GGATGATATCCAAAAGCTACAGG<br>TGTCCCACACCATTGCAG	0.99	95.45
AK358409	BLH4_UP22_L<br>BLH4_UP22_R	CTATCGCGGAGCCAAGTC<br>TTCATCTCCTCCGCGTACAT	0.99	101.88
AK375249	AN3_UP77_L<br>AN3_UP77_R	GATGTAGCGTCGGATGTCG<br>GCACTACATAGGGAGTGTTCACAA	0.98	102.03
AK375816	GA3ox2_UP25_L<br>GA3ox2_UP25_R	ACGACTACCGCCACTTCTGT<br>CAGCTTGTCGGCCAGAAC	0.99	102.45
AK357218	GA2ox10_UP88_<br>GA2ox10_UP88_	GCGTCGCTCTGCTCTTTC<br>GCCATCAGCTCCAGCAAC	0.97	97.34
MLOC_5515	CESa8_UP93_L<br>CESa8_UP93_R	CAGCTGCGCATCCTATCAG<br>ACTACCCTGCATCTGGCACT	0.99	97.36
AK366536	BLH1_UP92_L<br>BLH1_UP92_R	CAAGATCATGCTCGCCAAG<br>CTCGCGTTGATGAACCAGT	0.99	102.48
AK367579	BLH6_UP159_L<br>BLH6_UP159_R	ACCCGAAAGACTCGGAGAA<br>TGATGAACCAGTTCGACACC	0.99	100.3
MLOC_7323	HvCOMT2_UP9_<br>HvCOMT2_UP9_	TCGAAGCCCTTGTAGGACTC<br>AGGGATGAAGAACCACTCCA	0.99	100.05
MLOC_1605	GA20ox1_UP66_<br>GA20ox1_UP66_	TGGGATCCATCATGTCCTG<br>TCGAGGTCTTCTCCTCGTG	0.99	103.64
MLOC_6659	FAH1_UP73_L<br>FAH1_UP73_R	GATGTCTTATAGCGCTCTTGAGG<br>ACTCCTCCTTCCACCTATATAAACC	0.99	97.23
BM816519*	JIP23_F<br>JIP23_R	ATCACAGTGTGTGTGCAAAG<br>ACTTTTGCGCGTTAACATCC	0.98	100
X82937*	JIP37_F<br>JIP37_R	GATCCATCGACAAGAAGTCC<br>ACTGTGGGTCTTGAGCTTGT	0.93	94
BM815987*	JIP60_F<br>JIP60_R	CAGCAGCGACTTCATTTACA<br>ATGGTGTCGCAGACTATCCT	0.99	129
miR172^	RT-miR172a<br>Uni_MIRs<br>SLOmiR172	GGCGGAGAATCTTGATGATG<br>TGGTGCAGGGTCCGAGGTATT<br>GTCTCCTCTGGTGCAGGGTCCGAGGTATTCGCACcagagg agACATGCAG		

Development • Supplementary information

In situ hybridisation

Primer	Sequence
T7-H4-F	GTGTCATAATACGACTCACTATAGGATGTCAGGCCGTGGAAAG
H4-F	ATGTCAGGCCGTGGAAAG
T7-H4-R	GTGTCATAATACGACTCACTATAGGTTAACCACCAAATCCATATAGA GTCC
H4-R	TTAACCACCAAATCCATATAGAGTCC

UP, Universal Probe
*Davis (2011) PhD Thesis Glasgow University.

^Debenardti JM, Lin H, Chuck G, Faris JD and J Dubcovsky (2017) microRNA172 plays a crucial role in wheat spike morphogenesis and grain threshability. Development. 144(11): 1966–1975

Development • Supplementary information
